# Enhanced IgA coating of bacteria in women with *Lactobacillus crispatus-*dominated vaginal microbiota

**DOI:** 10.1186/s40168-021-01198-4

**Published:** 2022-01-24

**Authors:** Annelot C. Breedveld, Heleen J. Schuster, Robin van Houdt, Rebecca C. Painter, Reina E. Mebius, Charlotte van der Veer, Sylvia M. Bruisten, Paul H. M. Savelkoul, Marjolein van Egmond

**Affiliations:** 1 Department of Molecular Cell Biology and Immunology, Amsterdam Infection and Immunity Institute, Amsterdam UMC, Vrije Universiteit Amsterdam, De Boelelaan 1117, Amsterdam, 1081 HV The Netherlands; 2 Department of Medical Microbiology and Infection Control, Amsterdam Infection and Immunity Institute, Amsterdam UMC, Vrije Universiteit Amsterdam, Amsterdam, The Netherlands; 3 Department of Obstetrics and Gynecology, Amsterdam Reproduction and Development Institute, Amsterdam UMC, Amsterdam, University of Amsterdam, Meibergdreef 9, Amsterdam, 1105 AZ The Netherlands; 4 Department of Infectious Diseases, Amsterdam Infection and Immunity Institute, Public Health Service of Amsterdam (GGD), Nieuwe Achtergracht 100, Amsterdam, 1018 WT The Netherlands; 5 Department of Medical Microbiology, School of Nutrition and Translational Research in Metabolism (NUTRIM), Maastricht University Medical Center+, Maastricht, The Netherlands; 6 Department of Surgery, Amsterdam UMC, Vrije Universiteit Amsterdam, Amsterdam, The Netherlands

## Abstract

**Background:**

Immunoglobulin A (IgA) plays an important role in maintaining a healthy intestinal microbiome, but little is known about the interaction between local immunoglobulins and the vaginal microbiome. We assessed immunoglobulins (unbound and bound to bacteria), their association with vaginal microbiota composition and the changes over time in 25 healthy women of reproductive age.

**Results:**

In both *Lactobacillus crispatus*-dominated and non-*L. crispatus*-dominated microbiota, IgA and IgG (unbound and bound to bacteria) were higher during menses (*T* = 1) compared to day 7‑11 (*T* = 2) and day 17‑25 (*T* = 3) after menses onset. The majority of vaginal bacteria are coated with IgA and/or IgG. Women with *L. crispatus*-dominated microbiota have increased IgA coating of vaginal bacteria compared to women with other microbiota compositions, but contained less IgA per bacterium. Presence of a dominantly IgA-coated population at *T* = 2 and/or *T* = 3 was also strongly associated with *L. crispatus*-dominated microbiota. In women with non-*L. crispatus*-dominated microbiota, more bacteria were uncoated. Unbound IgA, unbound IgG, and bound IgG levels were not associated with microbiota composition.

**Conclusions:**

In conclusion, *L. crispatus*-dominated vaginal microbiota have higher levels of bacterial IgA coating compared to non-*L. crispatus*-dominated vaginal microbiota. Similar to its regulating function in the intestinal tract, we hypothesize that IgA is involved in maintaining *L. crispatus*-dominated microbiota in the female genital tract. This may play a role in *L. crispatus*-associated health benefits.

Video abstract

**Supplementary Information:**

The online version contains supplementary material available at 10.1186/s40168-021-01198-4.

## Background

A healthy vaginal mucosa is populated by a low diversity of species, generally dominated by either *Lactobacillus* species or diverse anaerobic bacteria. Of the *Lactobacillus* spp., *L. crispatus* and *L. iners* are the most prevalent in the vagina. Especially *L. crispatus*-dominated vaginal microbiota is considered more beneficial compared to *L. iners*-dominated or diverse vaginal microbiota since *L. crispatus* is associated with protection from pathogens and the regulation of anti-inflammatory responses [1–4]. A diverse vaginal microbiome is associated with bacterial vaginosis, the most common gynecological condition in women of reproductive age, and an increased risk of HIV, other sexually transmitted infections and preterm birth [5–7]. Furthermore, *L. crispatus*-dominated microbiota are less likely to shift to a diverse microbiome than *L. iners*-dominated vaginal microbiota [8–10]. *L. iners* is often found in diversified vaginal microbiota and therefore its potential beneficial role has been debated. The etiology of the different phenotypes of the distinct vaginal microbiota profiles is largely unknown.

In the intestinal tract, the interaction between microbiota and local immunoglobulins is important in maintaining mucosal homeostasis [11]. Immunoglobulin A (IgA) is the most abundant antibody present in the intestinal tract. It can be subdivided into IgA1 and IgA2, of which the latter is more resistant to proteolytic cleavage induced by bacterial proteases and is the most common subtype in the intestinal tract [12]. IgA can coat and contain the resident intestinal commensal microbiota (immune inclusion) and provide protection against enteric pathogens by inhibiting their entrance into the intestinal epithelium (immune exclusion) [13, 14]. As such, IgA contributes to the maintenance of a healthy and diversified microbiota composition [15, 16]. Vice versa, the microbiota also plays a significant role in regulating IgA levels [16–19]. Changes in immunoglobulin coating of intestinal bacteria have been associated with inflammatory bowel diseases [20, 21].

Unlike other mucosal tissues, IgG dominates IgA in the vaginal mucosa. Levels of both IgA and IgG vary during the menstrual cycle [22]. Little is known about the role of IgA and IgG in the female genital tract and knowledge regarding cross-talk between local immunoglobulins and vaginal microbiota is currently lacking. In this study, we investigated immunoglobulins (unbound and bound to bacteria) in relation to the vaginal microbiota composition and the influence of menses in women of reproductive age.

## Methods

### Study population

For this investigation we used microbiota data, meta data and samples from a previous study about the influence of vaginal douching on vaginal microbiota composition by van der Veer et al. [23]. The study protocol and methods were extensively described [21]. In short, women without sexually transmitted infections were recruited. Both hormonal contraceptive users and non-users were eligible. Participants were asked to self-collect vaginal swabs (155C Rayon dry swabs, Copan Diagnostics Inc., Murietta, CA) during the study. For the current study, only samples collected in the first month, when no vaginal douching was performed, were used. Participants collected a swab every other day. Swabs were stored at −20 °C at the Public Health Laboratory of Amsterdam. On average, eight swabs that were evenly distributed over the first month were selected per participant for microbiota composition analysis. Of these first month swabs, three swabs were selected for additional immunological analyses. The first of three swabs was collected on day three after onset of menstrual bleeding, the second between day seven and eleven after onset of menstrual bleeding and the third between day 17 and 25 after onset of menstrual bleeding. These specimens will be referred to as collected during menstrual bleeding or first time point (*T* = 1), second time point (7‑11 days after onset of menstrual bleeding; *T* = 2), and third time point (17‑25 days after onset of menstrual bleeding; *T* = 3) for all women.

### Profiling of immunoglobulins that were bound to bacteria

Vaginal swabs were washed in sterile phosphate-buffered saline (PBS; B.Braun, Melsungen, Germany) and vortexed for at least 10 s to extract bacteria from the rayon swabs. Samples were transferred to a 96-well v-bottom plate, centrifuged for 5 min (4000 rpm, 4 °C), and blocked with 5% endotoxin free bovine serum albumin (BSA) (Akron Biotech, Boca Raton, FL) for 20 min on ice. Bacteria were stained with F(ab′)2 anti-human IgA-AF647 (1:200) and F(ab′)2 anti-human IgG-AF488 (1:200) (both Jackson ImmunoResearch, West Grove, PA) for 30 min on ice. Samples were washed in PBS before flow cytometric analysis (Sony SH800S, Sony Biotechnology, San Jose, CA). For each sample, approximately 20,000 events were recorded.

FlowJo (version 10.6.2) was used for the analysis of the flow cytometry data. The coating index was calculated by multiplying the percentage of bacteria with bound immunoglobulin with the median fluorescence intensity (MFI). Contour plotting was used for cluster analysis of the flow cytometry data. The populations were defined by the appearance of a separate population in a contour plot and gates were manually set on the defined populations. A double negative population was defined by a MFI lower than the mean MFI plus three SD of the no stain samples for both IgA and IgG. A double positive population was defined by a MFI higher than the mean MFI plus three SD of the no stain samples for IgA or IgG. An IgA dominant population was defined by a more than three times higher IgA/IgG ratio compared to the double positive population.

### Quantification of unbound immunoglobulins

Total IgA, IgA1, IgA2, secretory IgA (SIgA), and IgG concentrations in vaginal swabs were determined by capture enzyme-linked immunosorbent assay (ELISA). Briefly, 96-well plates (Maxisorb Nalge Nunc International, Rochester, NY) were coated with 100 μL of the capture antibodies goat anti-human serum IgA (2 μg/ml, Jackson ImmunoResearch, West Grove, PA), mouse anti-human secretory component (1:10.000, Sigma-Aldrich Corporation, St. Louis, MO) or goat anti-human IgG (2 μg/ml, Invitrogen, Waltham, MA) in sodium carbonate buffer (15 mM NaCO3, pH 9,6) and incubated overnight at 4 °C. Nonspecific-binding free sites were blocked with 200 μl of blocking buffer (0.5% BSA in PBS containing 0.05% Tween20) for 60 min at 37 °C. Samples were thawed and bacterial content was spun (10.000 rpm, 7 min at 4 °C) after which the supernatant was diluted 1:20, 1:100 and 1:1000 in blocking buffer and 100 μl was incubated in duplicate for 60 min at 37 °C. Wells were incubated with 100 μl of detecting antibodies F(ab′)2 goat anti-human IgA-HRP (1:2000, Invitrogen, Waltham, MA), mouse anti-human IgA1-biotin (1:1000, Abcam, Cambridge, UK), mouse anti-human IgA2-biotin (1:1000, Abcam, Cambridge, UK) or F(ab′)2 goat anti-human IgG-HRP (1:2000, Invitrogen, Waltham, MA) and incubated for 60 min at 37 °C. For IgA1 and IgA2 detection, 100 μl of streptavidin poly-HRP (100 ng/ml, Sanquin, Amsterdam, The Netherlands) was added for 30 min at 37 °C. Presence of immunoglobulins was detected with 100 μl/well of 12 ml of 0,1M sodium acetate (NaAc pH 4) with 3 μl of 30% hydrogen peroxide and 200 μl of 6 mg/ml 3,3′,5,5′-tetramethylbenzidine (TMB). The reaction was stopped with 100 μl of sulfuric acid (10% H_2_SO_4_) and absorbance was measured with a microplate reader (Bio-Rad, Berkeley, CA) at 450 nm.

### Total protein concentration vaginal fluid

Total protein concentration of every vaginal swab sample was determined using Pierce^TM^ BCA Protein Assay Kits (ThermoFisher, Waltham, MA). In brief, 10 μL of vaginal fluid was added to a 96-well flat bottom plate, mixed with 200 μL of BCA working reagent, and incubated for 30 min at 37 °C. Absorbance was measured with a microplate reader (Bio-Rad, Berkeley, CA) at 562 nm when the plate was cooled to room temperature.

To correct for inter- and intra-participant variation, unbound immunoglobulin levels (defined with ELISA) were corrected for total protein content by dividing the measured immunoglobulin levels by total protein level and multiplied by a thousand (ng/1 mg protein). Immunoglobulin levels below detection limit (determined per 96-wells plate) were imputed ten times, and the average of the imputed levels were corrected for total protein content.

### Statistical analysis

Data were log-transformed to reach normality. We used repeated measures analyses (linear mixed models) with an unstructured variance-covariance matrix, to analyze the differences between time points with post hoc Bonferroni correction for individual comparisons. To analyze the effect of microbiota composition and participant characteristics on the measured outcomes, these variables were added individually as fixed parameters to the model. Spearman’s rho was used for correlation analysis. The Pearson chi-square test was used for categorical data. The paired *T* test was used for continuous data.

Spotfire (version 7.13.0) was used for creating heat maps and performing complete linkage hierarchical clustering. The linear discriminant analysis effect size (LEfSe) algorithm was used to identify bacterial taxa associated with changes in immunoglobulin-coated populations [24]. The median relative abundance of taxa at all available measurements was calculated and compared between participants with an IgA dominant population at time point two or three and participants who do not have a third population at any time point. LEfSe uses factorial Kruskal-Wallis rank-sum test to detect differential abundances of bacterial taxa between specified groups. The estimated effect size of the differentially abundant taxa was measured using linear discriminant analysis. An alpha-value of 0.01 for factorial Kruskal-Wallis test was considered significantly different between different classes. A minimum threshold of 2.0 was used for logarithmic latent discriminatory analysis score for discriminative features. Statistical analyses were performed using IBM SPSS statistics (version 26). Data of the immunoglobulin measurements were visualized using GraphPad Prism (version 8.2.1.).

## Results

### Study population

Vaginal swabs of twenty-five women were included in the current study. Participant characteristics are shown in Table 1. For extensive characteristics, see the original research paper [23]. The median age was 24 years (interquartile range (IQR): 22‑29). The majority of participants were of Dutch descent (*n* = 22, 88%). The other women had Eastern-European, Middle-Eastern, or European/African mixed heritage (*n* = 3, 12%). Smoking was reported by five women (20%). Fifteen women (60%) used oral hormonal contraceptives.

**Table 1:** Participant characteristics

Baseline characteristics	***n*** = 25 (%)
Age, years, median [IQR^a^]	24 [22‑29]
Ethnic background	
Dutch	22 (88)
Middle-Eastern	1 (4)
Eastern European	1 (4)
European/African (parental heritage)	1 (4)
Smoking	5 (20)
Oral contraceptive usage	15 (60)
Length of menstrual cycle, days, median [IQR]	28 [28‑28]
Protection during menses	
Tampons	18 (72)
Sanitary pads	2 (8)
Tampons and sanitary pads	4 (16)
Menstrual cup	1 (4)
Has ever been pregnant	1 (4)
Parity > 0	0 (0)

^a^*IQR* Interquartile range

### Immunoglobulins over time

**Fig. 1:**
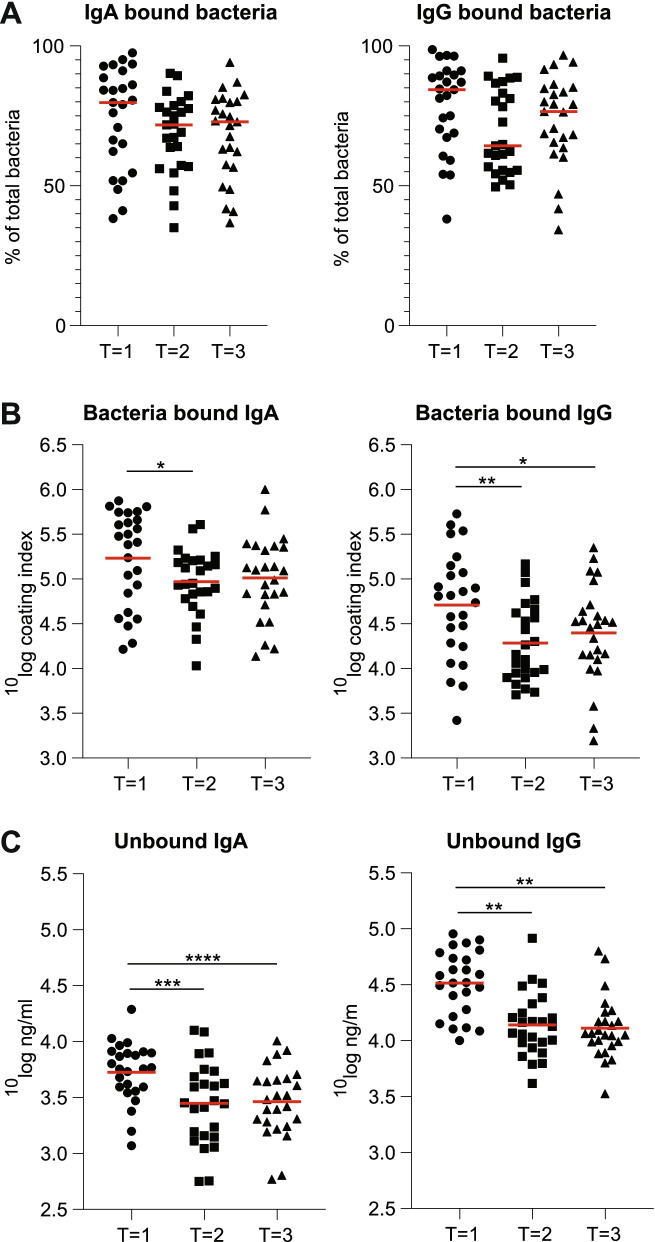
Bacteria with bound immunoglobulin, immunoglobulin bound to bacteria, and unbound immunoglobulins over time. Bacteria with bound immunoglobulin, immunoglobulin bound to bacteria and unbound immunoglobulins were measured during menstrual bleeding; time point 1 (*T* = 1), 7‑11 days after onset of menstrual bleeding; time point 2 (*T* = 2); and 17‑25 days after onset of menstrual bleeding; time point 3 (*T* = 3). **A** The percentage of bacteria with bound IgA or IgG over time. **B** Coating index of IgA and IgG bound to bacteria over time. **C** The concentration of unbound IgA and IgG over time. The red line represents the median for (**A**) and the mean for (**B**) and (**C**). *p < 0.05, **p < 0.01, ***p < 0.001, ****p < 0.0001

We investigated changes in immunoglobulins bound to bacteria and unbound immunoglobulins over time. Validation of our protocol and gating strategies can be found in Supplemental Material 1. The majority of bacteria are bound with both IgA and IgG at all time points, which is indicated as percentage of total Ig bound bacteria (Fig. 1A). The percentage of IgA or IgG bound bacteria did not significantly change over time. Bacteria bound IgA and IgG were quantified using the coating index (percentage immunoglobulin bound bacteria * MFI) and were highest during menses. Bound IgA was significantly increased during menses (*T* = 1) compared to the second time point (*T* = 2) (*p* = 0.044, Fig. 1B), while bound IgG was significantly increased at *T* = 1 compared to both *T* = 2 and the third time point (*T* = 3) (*p* = 0.006 and 0.042 respectively, Fig. 1B). Since the percentage of immunoglobulin bound bacteria was stable over time, the significant increase in IgA and IgG coating index was caused by an increase in MFI during menses. Correlation analysis revealed a statistically significant positive correlation between bound IgA and IgG at all time points (*T* = 1: Spearman’s rho = 0.74, *p* < 0.0001. *T* = 2: Spearman’s rho = 0.42, *p* = 0.038, *T* = 3: Spearman’s rho = 0.65, *p* = 0.0004, Fig. S1). The MFI per bacterium for both bacteria bound IgA and bacteria bound IgG was stable over all time points (Fig. S2).

In vaginal fluid, unbound total IgA and IgG increased significantly during *T* = 1 compared to *T* = 2 and *T* = 3 (*p* = 0.0003 and *p* < 0.0001 respectively for IgA, *p* < 0.0001 in both comparisons for IgG, Fig. 1C). IgA1 and IgA2 levels differed and showed contrasting patterns. IgA1 levels increased during *T* = 1 compared to *T* = 3 (*p* = 0.019, Fig. S3A), while IgA2 levels decreased during *T* = 1 compared to *T* = 3 (*p* = 0.002, Fig. S3B). SIgA levels showed a positive trend over time (similar to IgA2), but the differences between the time points were not statistically significant (Fig. S3C). The ratio between bound and unbound immunoglobulins over time was stable for both IgA and IgG (Fig. S4).

### Microbiota composition over time

We also investigated changes in vaginal microbiota over time. For most women, microbiota composition was stable during the study period (Fig. S5). In thirteen (52%) women, the same bacterium was dominant during all three time points. *L. crispatus* was dominant in eight (32%) and *L. iners* in five (20%) of these women. In six of the twelve women with changing microbiota profiles, the dominant bacterium switched between different *Lactobacillus* species. Of the remaining six women, one woman had a diverse profile at all time points and the other women switched between either a *Lactobacillus*-dominated profile or a profile dominated by *Gardnerella vaginalis*, *Megasphaera*, or *Bacterial Vaginosis-Associated Bacterium 1* (BVAB1).

**Fig. 2:**
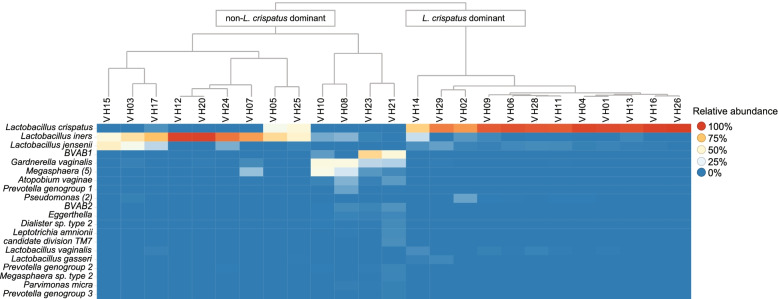
Vaginal microbiota composition. Heat map and clustering analysis. Heat map depicts the top 20 most abundant vaginal species in the 25 study participants. Colors reflect the median relative abundance of all available microbiota measurements during the menstrual cycle

Complete linkage hierarchical clustering was performed using median relative abundance of all available samples, shown as a heat map with dendrogram in Fig. 2. According to the hierarchical clustering, participants were divided in two groups, i.e., a *L. crispatus* dominated and a non-*L. crispatus*-dominated group. Twelve women with a *L. crispatus*-dominated vaginal microbiota composition clustered together. Women with other *Lactobacillus* spp. as dominant species (mainly *L. iners*) or with a diverse profile also clustered together, thirteen in total. The microbiota of nine women in the latter group was dominated by either *L. iners* or *L. jensenii* and in four cases the vaginal microbiota was diversified with low levels of *Lactobacillus* spp.

### Immunoglobulin coating of clustered microbiota

**Fig. 3:**
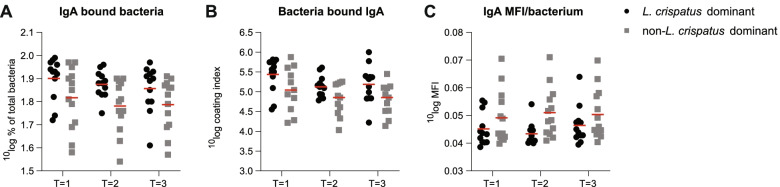
IgA bound bacteria, bacteria bound IgA, and IgA median fluorescence intensity (MFI) per bacterium in women with *L. crispatus*-dominated and non-*L. crispatus*-dominated microbiota over time. The **A** percentage of bacteria with bound IgA, **B** coating index of IgA bound to bacteria, and **C** IgA MFI per bacterium in women with *L. crispatus*-dominated vaginal microbiota compared to women having non-*L. crispatus*-dominated vaginal microbiota over time. Red line represents the mean

To estimate the overall effect of microbiota composition on bound and unbound immunoglobulins at all time points, a linear mixed effect model for repeated measurements was applied. Women with *L. crispatus*-dominated vaginal microbiota had on average a higher percentage of IgA bound bacteria (mean difference 0.08 ^10^log % 95% confidence interval (CI) 0.03‑0.14, *p* = 0.004, Fig. 3A) and a higher IgA-coating index (mean difference 0.33 ^10^log coating index 95% CI 0.11‑0.55, *p* = 0.005, Fig. 3B) compared to non-*L. crispatus*-dominated vaginal microbiota. In contrast, the IgA MFI per bacterium was lower in participants with *L. crispatus*-dominated microbiota (mean difference 0.0056 ^10^log MFI 95% CI 0.0015‑0.0097, *p* = 0.009, Fig. 3C). There was no statistically significant effect of microbiota composition on the percentage of IgG bound cells, IgG-coating index or IgG MFI per bacterium ratio using linear mixed model analysis (Fig. S6). No significant differences were found for any of the unbound immunoglobulins over time when comparing women with *L. crispatus*-dominated microbiota versus non-*L. crispatus*-dominated microbiota (Fig. S7). The ratio between bound and unbound IgA was higher in participants with *L. crispatus*-dominated microbiota (mean difference 0.10 95% CI 0.03‑0.18, *p* = 0.01, Fig. S8).

We evaluated whether participant characteristics influenced the effect of microbiota composition on bacteria bound and unbound immunoglobulins. We assessed the effect of smoking, sex partners in the last 6 months, vaginal *Candida albicans*, self-reported vaginal discharge, and hormonal contraceptive usage. None of these characteristics influenced the effect of microbiota composition on bacteria bound and unbound immunoglobulins. Some of these features had positive or negative associations with bound and unbound immunoglobulins (data not shown).

### Bacterial bound IgA and IgG clustering

**Fig. 4:**
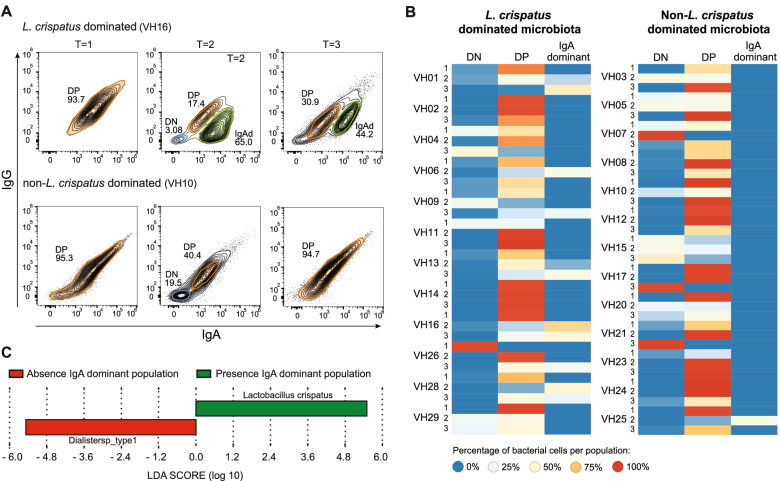
Bacterial clustering based on bacteria bound IgA and IgG. **A** Examples of contour plots of IgA- and IgG-coated bacterial populations. The double negative population (DN) is shown in blue, the double positive population (DP) is shown in orange and the IgA dominant (IgAd) is shown in green. Contour plots of a participant with *L. crispatus*-dominated vaginal microbiota (VH16) and a participant with non-*L. crispatus*-dominated vaginal microbiota (VH10). **B** Heat map depicting the percentage of bacterial cells per population (DN = double negative, DP = double positive and IgA dominant population). **C** The linear discriminant analysis (LDA) effect size analysis for the appearance of an IgA dominant population with an alpha-value set to 0.01. The linear discrimimant analysis (LDA) score for the taxon more prevalent in women with a IgA dominant population at *T* = 2 and/or *T* = 3 is shown in green, the LDA score for the taxon more prevalent in women without an IgA dominant population is shown in red

Bacterial immunoglobulin-coating analysis based on contour plotting revealed distinct IgA and IgG bound bacterial populations (Fig. 4A). Per participant, a maximum of three different populations was present: no bound IgA and IgG (double negative population, DN), bound IgA and IgG (double positive population, DP), and an IgA dominant population (IgA dominant population, IgAd). The contour plots of all participants are available in Supplemental Material 2, including a table with all MFI values per population. The percentages of bacteria per population per time point for each participant are shown in a heat map (Fig. 4B). For one participant (VH12) with two double positive populations, the percentages of both double positive populations were combined. Twenty-four samples (96%) contained a double positive population during menstrual bleeding. An IgA dominant bacterial population appeared in eight participants at the second or third time point. The IgA MFI was significantly increased and the IgG MFI was significantly decreased in the IgA dominant population compared to the double positive population (*p* = 0.001 for both comparisons). The IgA dominant population was strongly associated with *L. crispatus*-dominated microbiota (seven out of eight participants had *L. crispatus*-dominated microbiota, *p* = 0.007). One participant in the non-*L. crispatus*-dominated group, defined by hierarchical clustering, had an IgA dominant population at the second time point. Interestingly, at the second time point, the dominant species was *L. crispatus* (VH25, Fig. S1).

Subsequently, we investigated the effect of the individual taxa on the appearance of an IgA dominant population by performing LEfse. *L. crispatus*, as individual taxon, was strongly associated with the presence of an IgA dominant population (Fig. 4C). *Dialister* spp., commonly found in women with a diverse vaginal microbial profile, was strongly associated with the absence of an IgA dominant population.

There was no association between microbiota composition and the presence of a double negative population at separate time points, but there was an association with the size of the population. Participants with non-*L. crispatus*-dominated microbiota had more uncoated bacteria in their double negative population (mean difference 0.68 ^10^log % 95% CI 0.29‑1.07, *p* = 0.002, Fig. 4B double negative column).

## Discussion

In this explorative study, we describe the association between microbiota composition and immunoglobulins in the vagina. We combined flow cytometry and microbiota analyses to investigate host-microbe interactions in this mucosal niche. To our knowledge, this is the first report describing immunoglobulin coating of vaginal microbiota. We demonstrated that women with *L. crispatus*-dominated vaginal microbiota have increased IgA coating of vaginal bacteria compared to women with other microbiota compositions, but contained less IgA per bacterium. Women with non-*L. crispatus*-dominated microbiota had more unbound bacteria. IgG levels (unbound and bound to bacteria) did not associate with microbiota composition. Multiple sampling moments allowed us to investigate the influence of menstrual bleeding on immunoglobulins. We showed that coating of the vaginal microbiota with IgA and IgG was highest during menstrual bleeding compared to the other time points.

In the female genital tract, the role of IgA in regulating the vaginal microbiota composition is currently unknown. But there is evidence from the intestinal tract that IgA plays an important role in maintaining a healthy diversified microbiota composition. However, the exact mechanisms through which intestinal IgA promotes host-microbiota homeostasis remain unclear. It is thought that IgA influences bacterial metabolism which promotes diversification of intestinal commensals [18]. On the other side, metabolites produced by commensals, including short-chain fatty acids, can enhance IgA production in the intestinal tract [25]. To maintain homeostatic conditions, IgA is involved in both immune inclusion and immune exclusion [13]. Both processes are mediated by coating of commensals with IgA. Where immune exclusion is focused on keeping pathogens from moving across the mucosal barrier, immune inclusion refers to retaining commensals in the mucosa [13]. Anti-inflammatory and regulatory properties of mucosal dendritic cells in Peyer’s patches are enhanced by IgA-coated *L. rhamnosis* compared to uncoated *L. rhamnosis*, thereby contributing to a tolerogenic profile [26]. We hypothesize that IgA in the female genital tract, similar as in the intestinal tract, enhances a beneficial microbiota composition dominated by *L. crispatus*.

The IgA repertoire at mucosal sites is diverse, containing both specific and polyreactive IgA. In the gut, it is shown that T cell independent natural low-affinity polyreactive IgA binds non-invasive commensals, while more immunogenic and potential harmful commensals are bound by T cell dependent high-affinity IgA [15, 20, 27]. It is shown that the majority of *Lactobacillus* spp. in the intestinal tract is coated with IgA [20]. In mouse studies, colonization of the intestinal tract with *Lactobacillus* spp. did not mount a specific SIgA response [28]. Specific IgA against pathogens can be elicited in the female genital tract, for example, against virulence factors from *G. vaginalis*, a bacterium commonly present in women with diverse microbiota [29–31]. In a mouse model for *Chlamydia*, specific IgA is present which protects against *Chlamydia* infection [32]. In our study, we were not able to distinguish between polyreactive and specific immunoglobulins. Women with *L. crispatus*-dominated microbiota had higher IgA coating overall, but lower IgA coating per bacterium compared to women with non-*L. crispatus*-dominated microbiota. An explanation could be that the IgA bound to bacteria in *L. crispatus*-dominated microbiota has a relative low affinity. T cell independent low-affinitiy polyreactive IgA could be the predominant type bound to bacteria in *L. crispatus*-dominated microbiota.

The higher levels of unbound bacteria in non-*L. crispatus*-dominated microbiota could be relevant for increased inflammation seen in women with diverse microbiota. Several studies demonstrated that women with dysbiosis or bacterial vaginosis have increased levels of pro-inflammatory cytokines, without clinical evidence of inflammation [33, 34]. In vitro models showed that bacteria associated with dysbiosis, such as *Prevotella* spp. and *Atopobium vaginae*, elicited pro-inflammatory immune response when compared to *L. crispatus* [35–37]. It is unknown how this relates to the production of local immunoglobulins, but it suggests that bacteria associated with dysbiosis are more immunogenic in comparison with vaginal commensal lactobacilli. As IgA coating of bacteria has anti-inflammatory properties, the presence of more uncoated bacteria in non-*L. crispatus*-dominated microbiota could have a more pro-inflammatory effect. Because the group of participants with non-*L. crispatus*-dominated microbiota in our study mainly consisted of women with *L. iners*-dominated microbiota, this hypothesis should be tested in a larger study with more women with diverse vaginal microbiota.

Preservation of stable *L. crispatus*-dominated vaginal microbiota is beneficial for the host’ health. Women with *L. crispatus*-dominated microbiota are less likely to shift to a diverse microbiota composition and are less vulnerable to infections [8]. In this study, most women had stable vaginal microbiota composition over time. This is in line with previous studies in which the majority of healthy natural cycling women had stable vaginal microbiota compositions during their menstrual cycle, with only modest fluctuations in species richness [38, 39]. However, it was also shown that vaginal diversity can reversibly change during menstrual bleeding with a 100-fold decrease in *L. crispatus* and an increase in bacterial vaginosis-associated species including *L. iners*, *Gardnerella vaginalis*, *Prevotella bivia*, and *Atopobium vaginae* [9, 40, 41]. Most women return to a favorable Lactobacillus-dominated microbiota after menstrual bleeding which strengthens the view that presence of blood affects the vaginal microbiota composition temporarily. The fact that *G. vaginalis* can use hemoglobin as growth factor supports this hypothesis [40]. It remains unclear what causes the difference between a bacterial vaginosis-associated microbiota composition during menstrual bleeding and the clinical condition bacterial vaginosis. Discrepancies found between studies investigating the vaginal microbiota composition might be explained by the amount and duration of blood loss during menses, sexual activity and change of sexual partner, ethnicity, and many other factors. In our study, we found associations between several participant characteristics and bound and unbound immunoglobulins. It is important to take into account that individual participant features can influence microbiota composition and immunological aspects present in the vaginal mucosa.

Unlike in the intestinal tract, IgG dominates over IgA in the vagina and levels vary over the reproductive cycle [22]. Variations in immunoglobulin levels can be explained by several factors including the production rate of immunoglobulins, transport capacity of immunoglobulins and the origin of immunoglobulins at certain points during the cycle [42]. Previous studies showed fluctuations in unbound IgA and IgG levels in naturally cycling women, while oral contraceptive users contained more stable unbound IgA and IgG levels [22, 43, 44]. We found that vaginal IgG and IgA levels were highest during menstrual bleeding, which is consistent with existing literature [22, 45]. The increased total IgA and IgG levels during menstrual bleeding most likely derive from menstrual blood, as serum contains high levels of IgG (8‑14 mg/ml) and IgA (2‑3 mg/ml) [46]. Even though the variation of menstrual cycle was limited in our study, menstrual length can last up to 35 days, mainly due to increased length of the follicular phase [47]. This might indicate that women with an increased follicular phase also have longer menses. As such, these women might be exposed longer to higher IgG and IgA levels and possible changes in microbiota composition. Our findings are consistent with the concept that the presence of menstrual blood in the vagina plays an important role in modulation of local immunoglobulin concentrations.

A limitation of this study is the sample size. Although this is a longitudinal study, the number of women included is relatively small. The enhanced IgA coating of bacteria observed in women with *L. crispatus* dominant communities included in this pilot study, should be interpreted with care. In this group of women, *L. crispatus*-dominated vaginal microbiota have higher levels of bacterial IgA coating compared to non-*L. crispatus*-dominated vaginal microbiota. However, to what extent and what the implications are remains unclear. Therefore, this study should be repeated with a larger sample size and with more variation in microbiota compositions.

## Conclusions

In conclusion, *L. crispatus*-dominated microbiota is associated with enhanced bacterial IgA coating. We propose that IgA in the vagina is involved in regulating the maintenance of stable microbiota in which *L. crispatus* dominates, similar to its function in the intestinal tract. Further research, including more women, is needed to test this hypothesis.

## Supplementary Information

**Additional file 1: Figure S1.** Correlation between bacteria bound IgA and IgG. Correlation between bacteria bound IgA and IgG during menstrual bleeding; time point 1 (T = 1), 7-11 days after onset of menstrual bleeding; time point 2 (T = 2) and 17-25 days after onset of menstrual bleeding; time point 3 (T = 3). Data visualized as ^10^log coating index (CI, coating index = percentage of immunoglobulin bound bacteria * median fluorescence intensity).

**Additional file 2: Figure S2.** Median Fluorescence Intensity (MFI) per bacterium. The MFI of immunoglobulins bound to bacteria was measured during menstrual bleeding; time point 1 (T = 1), 7-11 days after onset of menstrual bleeding; time point 2 (T = 2) and 17-25 days after onset of menstrual bleeding; time point 3 (T = 3) and divided by the percentage of bacteria with bound IgA or IgG. Red line represents the median.

**Additional file 3: Figure S3.** Immunoglobulin levels in vaginal fluid over time. Unbound immunoglobulins were measured menstrual bleeding; time point 1 (T = 1), 7-11 days after onset of menstrual bleeding; time point 2 (T = 2) and 17-25 days after onset of menstrual bleeding; time point 3 (T = 3). The level of (A) unbound IgA1, (B) unbound IgA2 and (C) unbound SIgA over time. All data visualized as ^10^log of the unbound immunoglobulin concentration corrected for total protein. Red line represents the mean. * *p* < 0.05 ** *p* < 0.01.

**Additional file 4: Figure S4.** The ratio between bound and unbound immunoglobulins. Bound and unbound immunoglobulins were measured during menstrual bleeding; time point 1 (T = 1), 7-11 days after onset of menstrual bleeding; time point 2 (T = 2) and 17-25 days after onset of menstrual bleeding; time point 3 (T = 3). Bound immunoglobulins (coating index) were divided by unbound immunoglobulins (concentration) to calculate the ratio. Red line represents the mean.

**Additional file 5: Figure S5.** Microbiota composition over time. Heat map depicting the top 20 most abundant species among the 25 study participants during menstrual bleeding (1), time point 2 (2) and time point 3 (3). Colors reflect the relative abundance.

**Additional file 6: Figure S6.** Bacteria with bound IgG, IgG bound to bacteria and IgG Median Fluorescence Intensity (MFI) per bacterium in women with *L. crispatus* dominated and non-*L. crispatus* dominated microbiota over time. The (A) percentage of bacteria with bound IgG, (B) coating index of IgG bound to bacteria and (C) IgG MFI per bacterium in women with *L. crispatus* dominated vaginal microbiota compared to women having non-*L. crispatus* dominated vaginal microbiota over time. Red line represents the mean.

**Additional file 7: Figure S7.** Immunoglobulin levels in vaginal fluid from women with *L. crispatus* dominated and non-*L. crispatus* dominated microbiota over time. Unbound IgA, IgG, IgA1, IgA2 and SIgA in women with *L. crispatus* dominated vaginal microbiota compared to women having non-*L. crispatus* dominated vaginal microbiota over time. Data visualized as ^10^log of the unbound immunoglobulin concentration corrected for total protein. Red line represents the mean.

**Additional file 8: Figure S8.** The ratio between bound and unbound immunoglobulins in women with *L. crispatus* dominated and non-*L. crispatus* dominated microbiota over time. Bound and unbound immunoglobulins were measured menstrual bleeding; time point 1 (T = 1), 7-11 days after onset of menstrual bleeding; time point 2 (T = 2) and 17-25 days after onset of menstrual bleeding; time point 3 (T = 3) and divided to calculate the ratio. These ratios were compared between women with *L. crispatus* dominated vaginal microbiota and women having non-*L. crispatus* dominated vaginal microbiota over time. Red line represents the mean.

**Additional file 9. Supplemental material 1.** Validation of our protocol and gating strategies.

**Additional file 10. Supplemental material 2.** Contour plots of all participants including a table with all MFI values per population. DN = double negative population, DP = double positive population, IgA dom = IgA dominant population.

## Data Availability

The data from the original study is available [doi: 10.1186/s12866-019-1545-0]. The microbiota dataset used for this study is available from C. van der Veer (email: charlottevdveer@gmail.com) on reasonable request. The data generated and analyzed during the current study are not publicly available because the data are accessory to the original study, but are available from the corresponding author and C. van der Veer on reasonable request.

## Abbreviations

BCA: Bicinchoninic acid assay
BVAB: *Bacterial Vaginosis-Associated Bacterium*
ELISA: Enzyme-linked immunosorbent assay
HIV: Human immunodeficiency virus
HRP: Horseradish peroxidase
IgA: Immunoglobulin A
IgG: Immunoglobulin B
IQR: Interquartile range
LEfse: Linear discriminant analysis effect size
MFI: Median fluorescence intensity
PBS: Phosphate-buffered saline
SIgA: Secretory IgA
