# Supplementary figures and images for: Enhanced IgA coating of bacteria in women with Lactobacillus crispatus-dominated vaginal microbiota
Source: Microbiome. 2022 Jan 24;10:15. doi: 10.1186/s40168-021-01198-4 (PMC8787895; doi:10.1186/s40168-021-01198-4)

**T=1**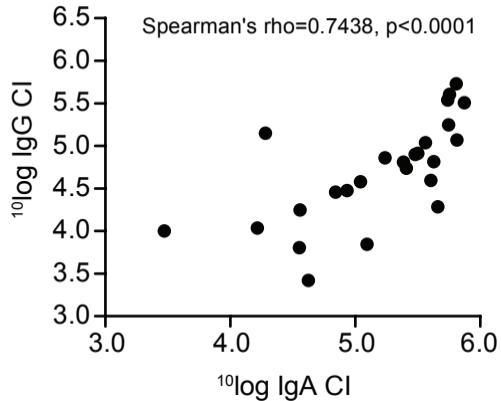**T=2**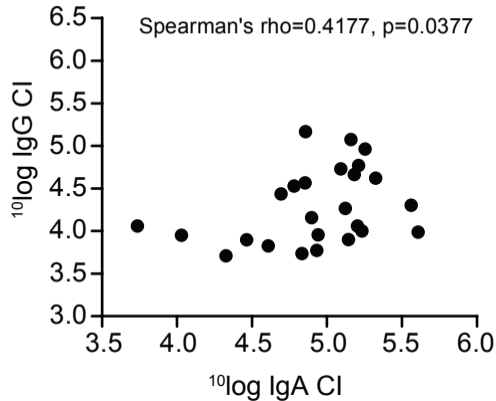**T=3**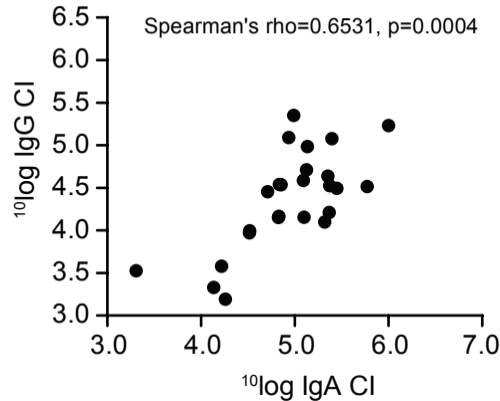

Supplement: Supplementary file 2 — Additional file 1: Figure S1. Correlation between bacteria bound IgA and IgG. Correlation between bacteria bound IgA and IgG during menstrual bleeding; time point 1 (T = 1), 7-11 days after onset of menstrual bleeding; time point 2 (T = 2) and 17-25 days after onset of menstrual bleeding; time point 3 (T = 3). Data visualized as 10log coating index (CI, coating index = percentage of immunoglobulin bound bacteria * median fluorescence intensity). [file 40168_2021_1198_MOESM2_ESM.pdf]

**IgA MFI/bacterium**

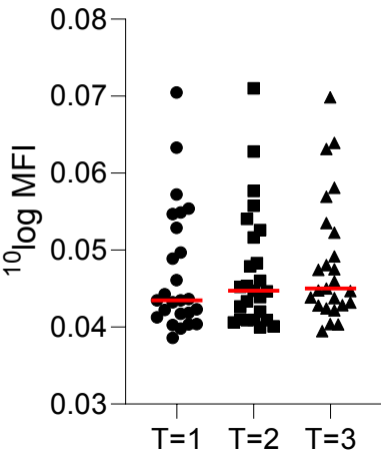

**IgG MFI/bacterium**

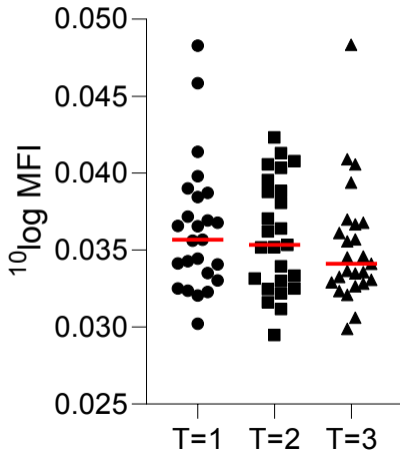

Supplement: Supplementary file 3 — Additional file 2: Figure S2. Median Fluorescence Intensity (MFI) per bacterium. The MFI of immunoglobulins bound to bacteria was measured during menstrual bleeding; time point 1 (T = 1), 7-11 days after onset of menstrual bleeding; time point 2 (T = 2) and 17-25 days after onset of menstrual bleeding; time point 3 (T = 3) and divided by the percentage of bacteria with bound IgA or IgG. Red line represents the median. [file 40168_2021_1198_MOESM3_ESM.pdf]

**A****Unbound IgA1**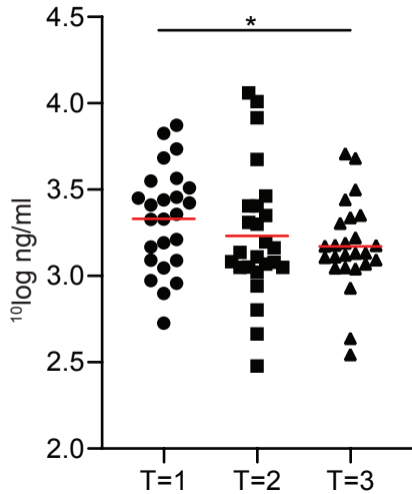**B****Unbound IgA2**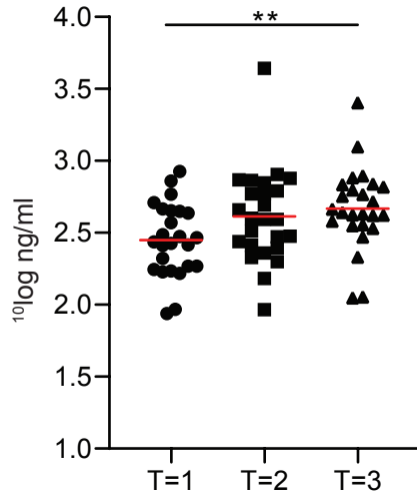**C****Unbound SIgA**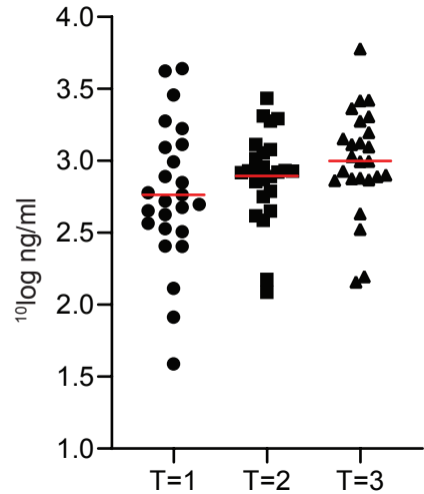

Supplement: Supplementary file 4 — Additional file 3: Figure S3. Immunoglobulin levels in vaginal fluid over time. Unbound immunoglobulins were measured menstrual bleeding; time point 1 (T = 1), 7-11 days after onset of menstrual bleeding; time point 2 (T = 2) and 17-25 days after onset of menstrual bleeding; time point 3 (T = 3). The level of (A) unbound IgA1, (B) unbound IgA2 and (C) unbound SIgA over time. All data visualized as 10log of the unbound immunoglobulin concentration corrected for total protein. Red line represents the mean. * p < 0.05 ** p < 0.01. [file 40168_2021_1198_MOESM4_ESM.pdf]

**Bound/unbound IgA**

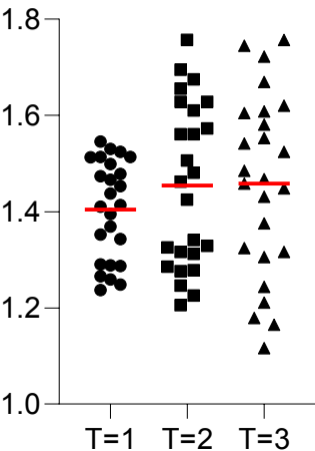

**Bound/unbound IgG**

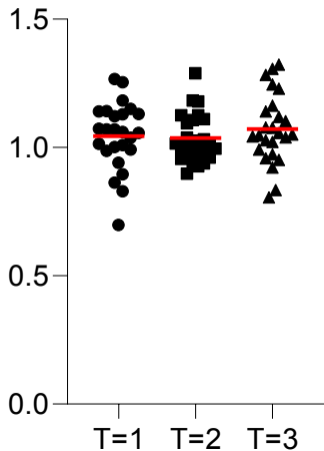

Supplement: Supplementary file 5 — Additional file 4: Figure S4. The ratio between bound and unbound immunoglobulins. Bound and unbound immunoglobulins were measured during menstrual bleeding; time point 1 (T = 1), 7-11 days after onset of menstrual bleeding; time point 2 (T = 2) and 17-25 days after onset of menstrual bleeding; time point 3 (T = 3). Bound immunoglobulins (coating index) were divided by unbound immunoglobulins (concentration) to calculate the ratio. Red line represents the mean. [file 40168_2021_1198_MOESM5_ESM.pdf]

## participants

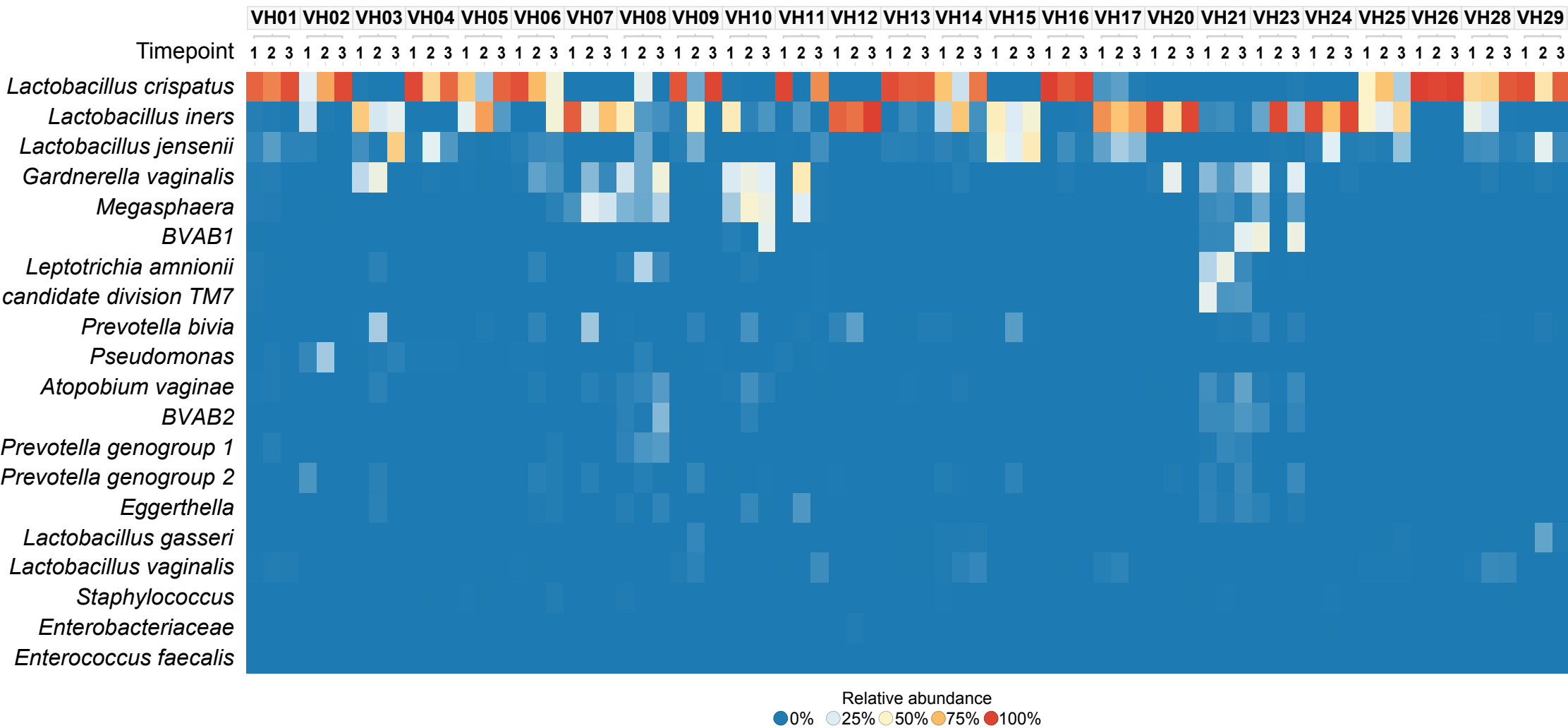

Supplement: Supplementary file 6 — Additional file 5: Figure S5. Microbiota composition over time. Heat map depicting the top 20 most abundant species among the 25 study participants during menstrual bleeding (1), time point 2 (2) and time point 3 (3). Colors reflect the relative abundance. [file 40168_2021_1198_MOESM6_ESM.pdf]

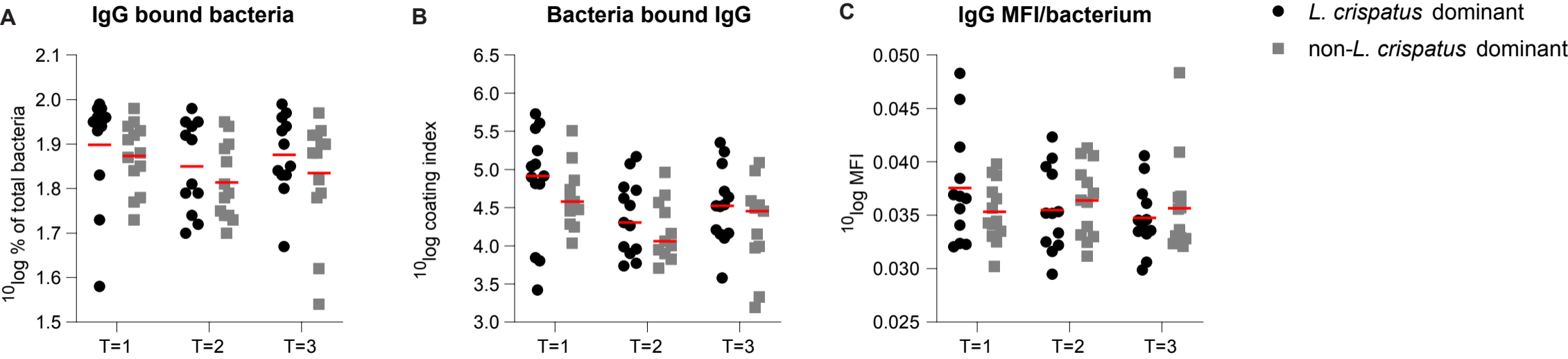

Supplement: Supplementary file 7 — Additional file 6: Figure S6. Bacteria with bound IgG, IgG bound to bacteria and IgG Median Fluorescence Intensity (MFI) per bacterium in women with L. crispatus dominated and non-L. crispatus dominated microbiota over time. The (A) percentage of bacteria with bound IgG, (B) coating index of IgG bound to bacteria and (C) IgG MFI per bacterium in women with L. crispatus dominated vaginal microbiota compared to women having non-L. crispatus dominated vaginal microbiota over time. Red line represents the mean. [file 40168_2021_1198_MOESM7_ESM.pdf]

**Bound/unbound IgA**

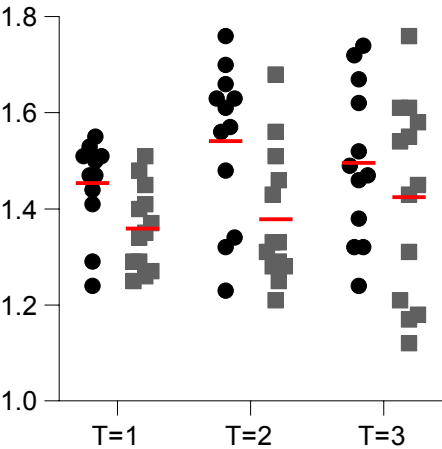

**Bound/unbound IgG**

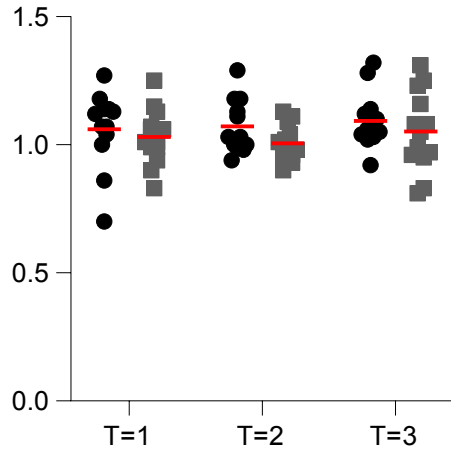

● *L. crispatus* dominant

■ non-*L. crispatus* dominant

Supplement: Supplementary file 9 — Additional file 8: Figure S8. The ratio between bound and unbound immunoglobulins in women with L. crispatus dominated and non-L. crispatus dominated microbiota over time. Bound and unbound immunoglobulins were measured menstrual bleeding; time point 1 (T = 1), 7-11 days after onset of menstrual bleeding; time point 2 (T = 2) and 17-25 days after onset of menstrual bleeding; time point 3 (T = 3) and divided to calculate the ratio. These ratios were compared between women with L. crispatus dominated vaginal microbiota and women having non-L. crispatus dominated vaginal microbiota over time. Red line represents the mean. [file 40168_2021_1198_MOESM9_ESM.pdf]
