# Supplementary material for: Enhanced IgA coating of bacteria in women with Lactobacillus crispatus-dominated vaginal microbiota
Source: Microbiome. 2022 Jan 24;10:15. doi: 10.1186/s40168-021-01198-4 (PMC8787895; doi:10.1186/s40168-021-01198-4)
Supplement: Supplementary file 8 — Additional file 7: Figure S7. Immunoglobulin levels in vaginal fluid from women with L. crispatus dominated and non-L. crispatus dominated microbiota over time. Unbound IgA, IgG, IgA1, IgA2 and SIgA in women with L. crispatus dominated vaginal microbiota compared to women having non-L. crispatus dominated vaginal microbiota over time. Data visualized as 10log of the unbound immunoglobulin concentration corrected for total protein. Red line represents the mean. [file 40168_2021_1198_MOESM8_ESM.pdf]

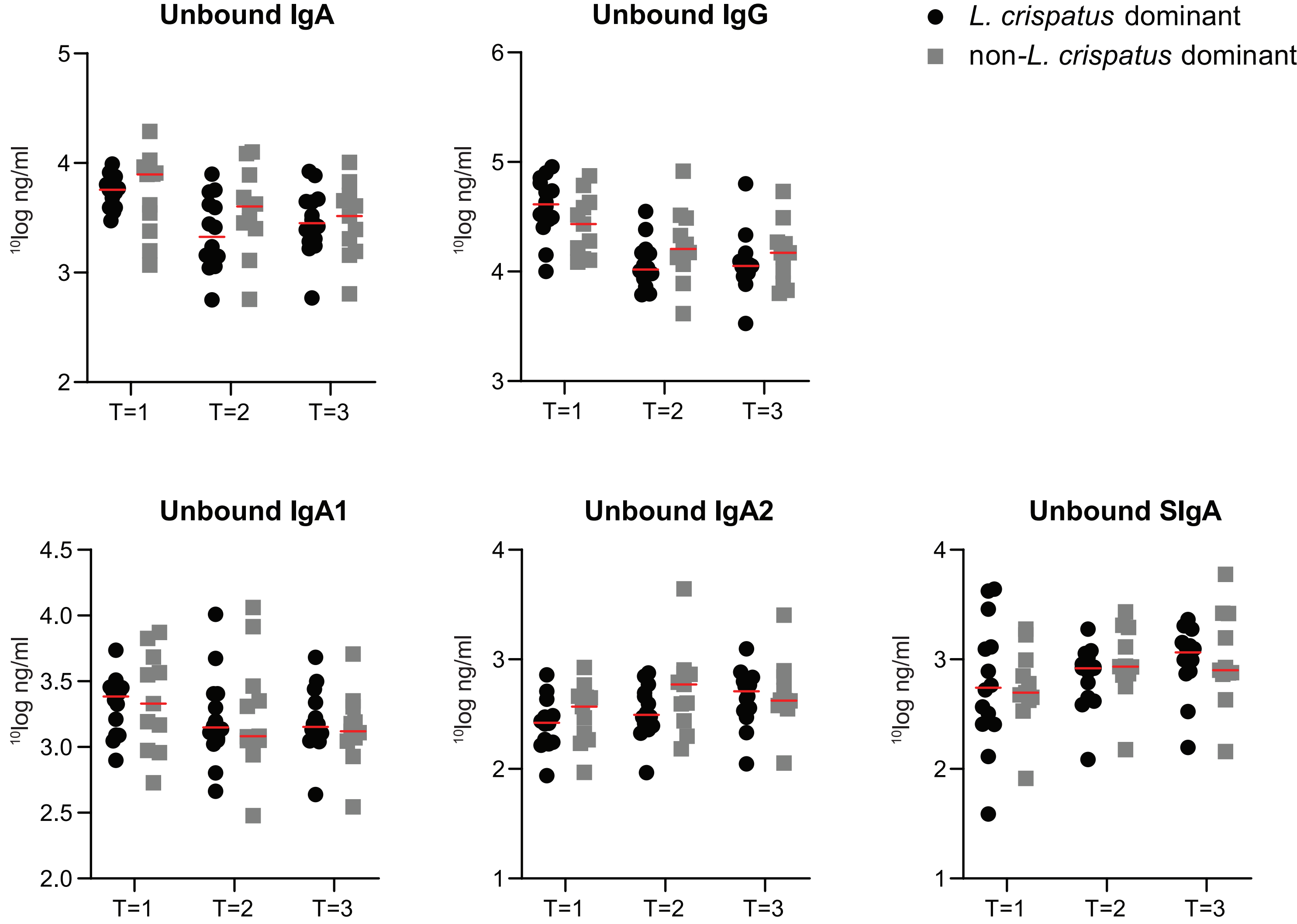

Unbound IgA
Unbound IgG
L. crispatus dominant
non-L. crispatus dominant
Unbound IgA1
Unbound IgA2
Unbound SIgA
10log ng/ml
T=1
T=2
T=3
