# Supplementary material for: Enhanced IgA coating of bacteria in women with Lactobacillus crispatus-dominated vaginal microbiota
Source: Microbiome. 2022 Jan 24;10:15. doi: 10.1186/s40168-021-01198-4 (PMC8787895; doi:10.1186/s40168-021-01198-4)
Supplement: Supplementary file 10 — Additional file 9. Supplemental material 1. Validation of our protocol and gating strategies. [file 40168_2021_1198_MOESM10_ESM.docx]

**Supplemental material 1**

**Supplemental methods**

Flow cytometric analysis with Syto9

Vaginal swabs from control persons with similar storage conditions were used. Additional validation methods were performed using the same protocol as described in the main article. Syto9 (Thermofisher, Waltham, MA) stain was performed according to the manufacturer’s protocol, using a concentration of 20µM. Flow cytometric analysis was performed with LSRFortessa™ X-20 (BD Biosciences).

**Supplemental results**

Validation of flow cytometric analysis using Syto9

To differentiate between noise from the flow cytometer and bacteria we used Syto9, a stain that binds DNA and is permeant to cell membranes. We used the forward (FSC) and side scatter (SSC) channels to set a gate to separate the bacteria from the noise. Because of incompatibility of Syto9 with the other fluorescent antibodies used, we set a cut-off in advance of 95% Syto9-positive cells within this gate to make Syto9 staining redundant. Vaginal swabs from five different participants showed an average of 95.9% (Standard Deviation (SD) 0.8) Syto9-positive cells within the gate, compared to 92.8% (SD 1.3) Syto9-positive cells ungated. An example is depicted in **figure SM1**. The ‘bacteria’ gate as shown in figure SM1 B was applied to all samples.


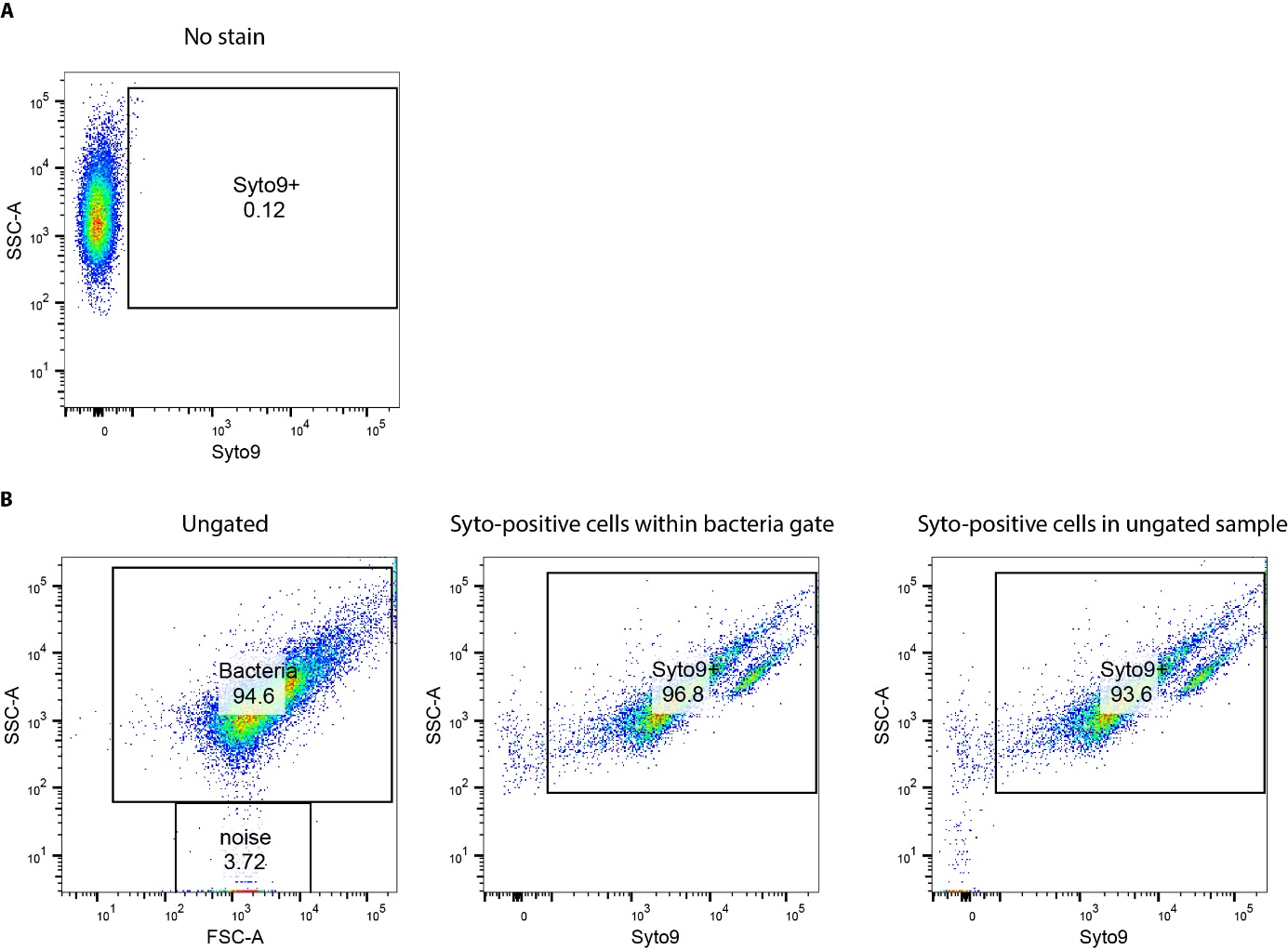


**Figure SM1. Syto9 staining of bacteria from vaginal swabs.** **(A)** Unstained vaginal swab to reveal auto-fluorescence of vaginal bacteria. **(B)** Vaginal swab stained with Syto9 . Data are representative for n=5.

Gating strategy for bacteria bound immunoglobulins

Gating for IgA and IgG measurements were set using samples without any staining. With these gates for bacteria with bound IgA and IgG, we distinguished bacteria without bound IgA and IgG from bacteria with bound IgA and/or IgG (total Ig bound bacteria). An example of the gating strategy used is shown in **figure SM2.**


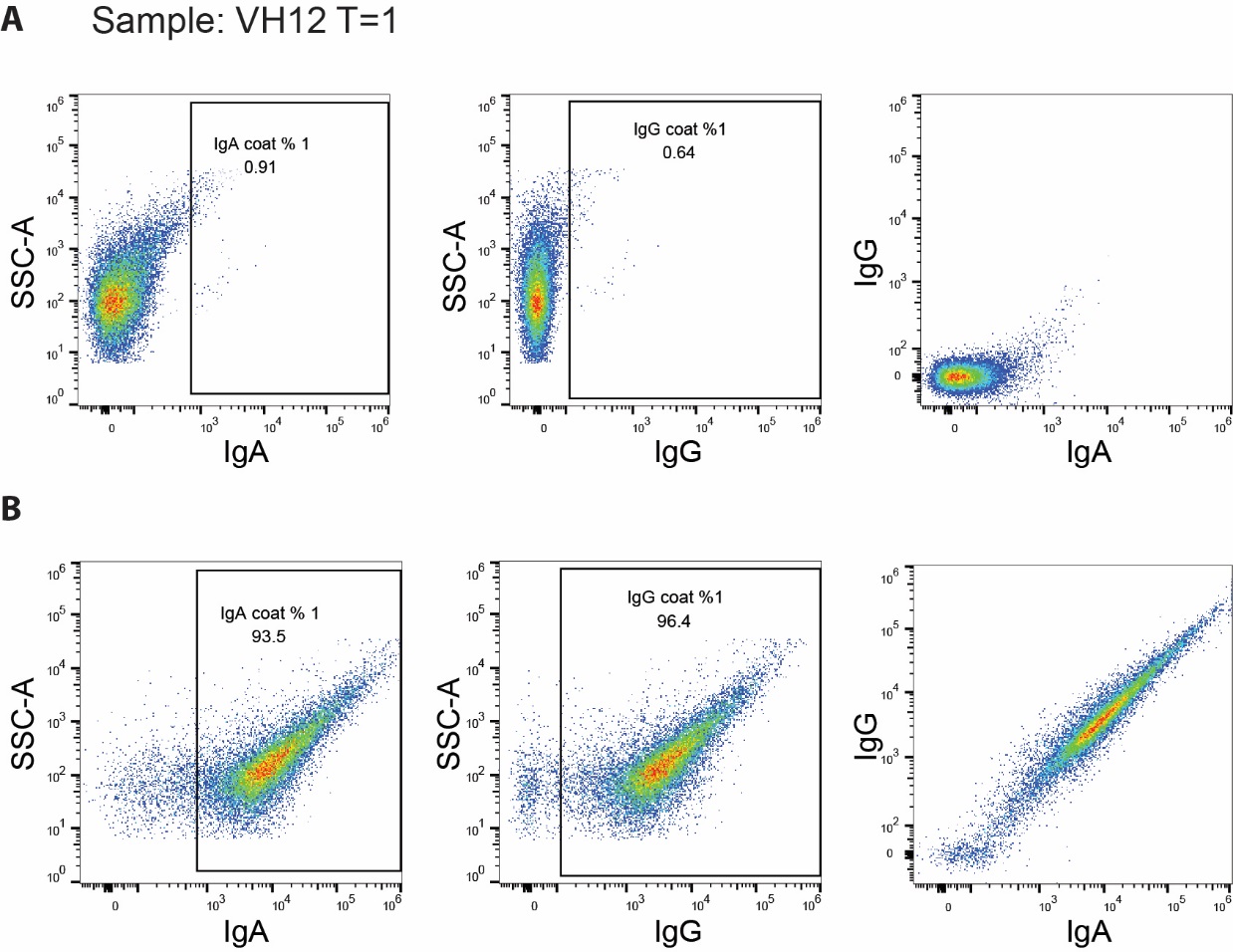


**Figure SM2. Gating strategy.** The gating strategy is shown for an example sample, from participant VH12, first time point (T=1). **(A)** samples without IgA or IgG staining were used to determine the gating limits for bacteria with bound IgA (left), bound IgG (middle) and without bound IgA or IgG (right, Q4). **(B)** bacteria with bound IgA (left), bound IgG (middle) and without bound IgA or IgG (right, Q4) of a sample with staining for IgA and IgG.
