# Supplementary material for: Enhanced IgA coating of bacteria in women with Lactobacillus crispatus-dominated vaginal microbiota
Source: Microbiome. 2022 Jan 24;10:15. doi: 10.1186/s40168-021-01198-4 (PMC8787895; doi:10.1186/s40168-021-01198-4)
Supplement: Supplementary file 11 — Additional file 10. Supplemental material 2. Contour plots of all participants including a table with all MFI values per population. DN = double negative population, DP = double positive population, IgA dom = IgA dominant population. [file 40168_2021_1198_MOESM11_ESM.pdf]

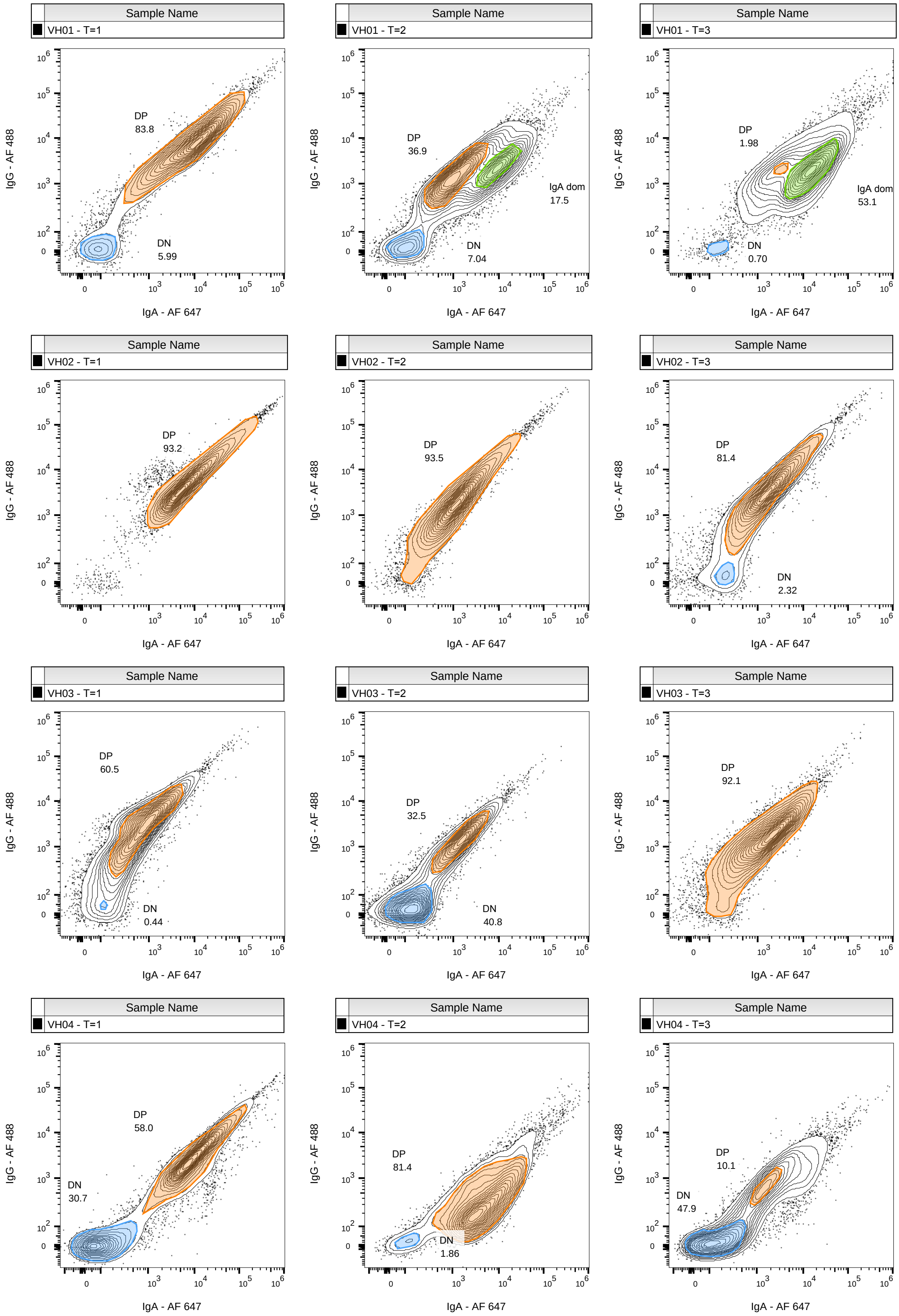
Sample Name
VH01 - T=1
DP
83.8
DN
5.99
Sample Name
VH01 - T=2
DP
36.9
IgA dom
17.5
DN
7.04
Sample Name
VH01 - T=3
DP
1.98
IgA dom
53.1
DN
0.70
Sample Name
VH02 - T=1
DP
93.2
Sample Name
VH02 - T=2
DP
93.5
Sample Name
VH02 - T=3
DP
81.4
DN
2.32
Sample Name
VH03 - T=1
DP
60.5
DN
0.44
Sample Name
VH03 - T=2
DP
32.5
DN
40.8
Sample Name
VH03 - T=3
DP
92.1
Sample Name
VH04 - T=1
DP
58.0
DN
30.7
Sample Name
VH04 - T=2
DP
81.4
DN
1.86
Sample Name
VH04 - T=3
DP
10.1
DN
47.9
IgG - AF 488
IgA - AF 647

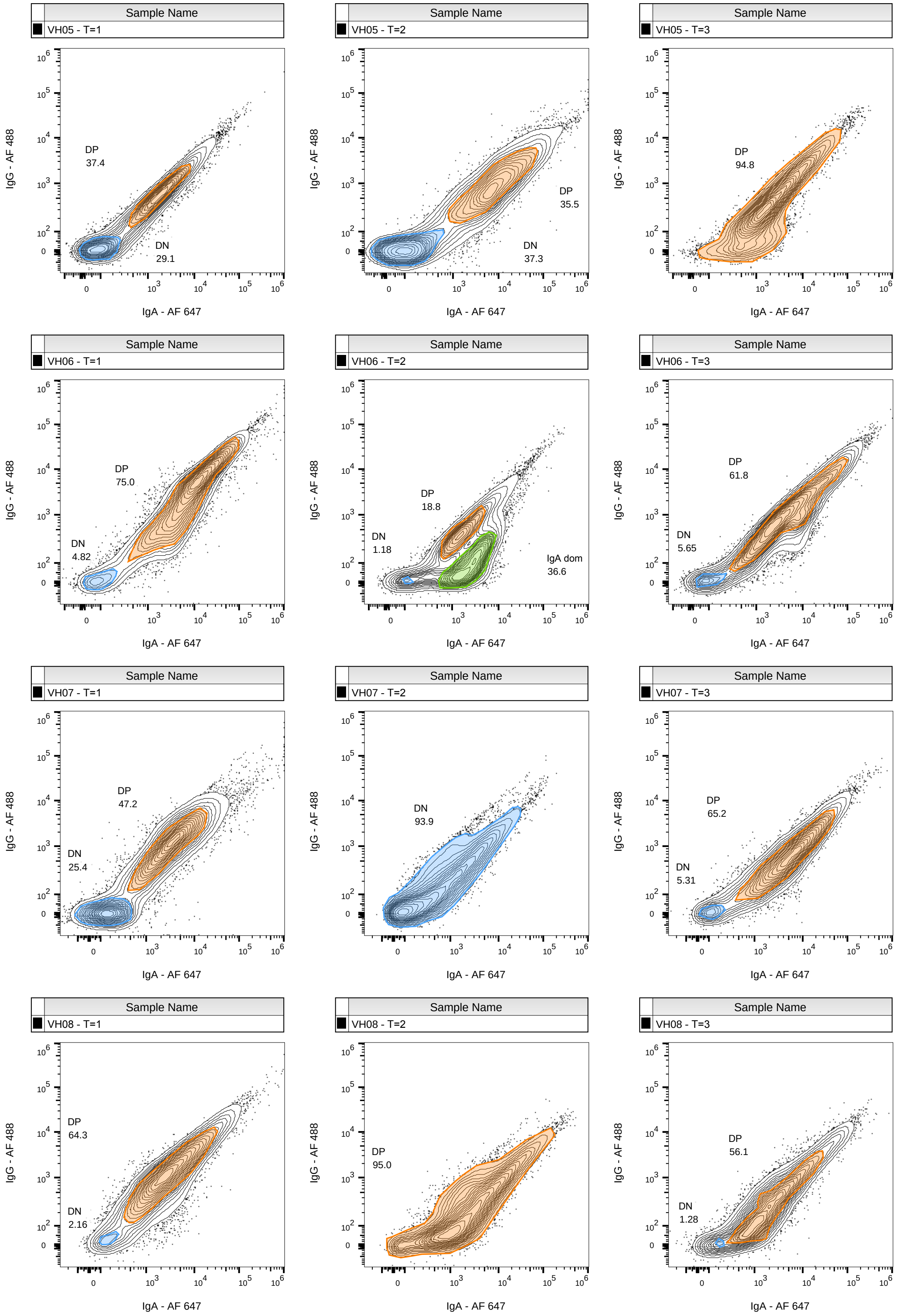

Sample Name
VH05 - T=1
DP
37.4
DN
29.1
Sample Name
VH05 - T=2
DP
35.5
DN
37.3
Sample Name
VH05 - T=3
DP
94.8
Sample Name
VH06 - T=1
DP
75.0
DN
4.82
Sample Name
VH06 - T=2
DP
18.8
DN
1.18
IgA dom
36.6
Sample Name
VH06 - T=3
DP
61.8
DN
5.65
Sample Name
VH07 - T=1
DP
47.2
DN
25.4
Sample Name
VH07 - T=2
DN
93.9
Sample Name
VH07 - T=3
DP
65.2
DN
5.31
Sample Name
VH08 - T=1
DP
64.3
DN
2.16
Sample Name
VH08 - T=2
DP
95.0
Sample Name
VH08 - T=3
DP
56.1
DN
1.28
IgG - AF 488
IgA - AF 647

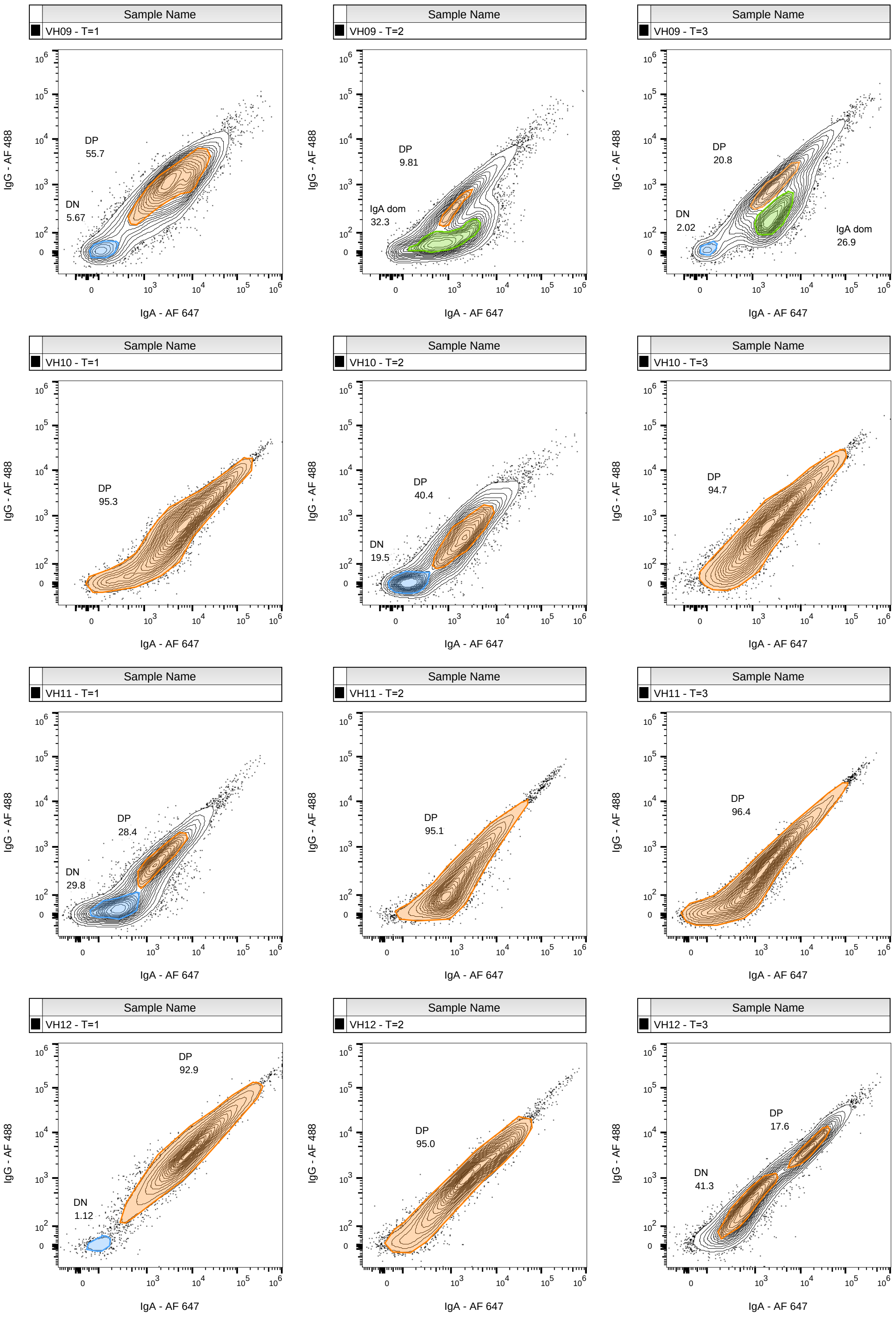
Sample Name
VH09 - T=1
DP
55.7
DN
5.67
IgG - AF 488
IgA - AF 647
Sample Name
VH09 - T=2
DP
9.81
IgA dom
32.3
IgG - AF 488
IgA - AF 647
Sample Name
VH09 - T=3
DP
20.8
DN
2.02
IgA dom
26.9
IgG - AF 488
IgA - AF 647
Sample Name
VH10 - T=1
DP
95.3
IgG - AF 488
IgA - AF 647
Sample Name
VH10 - T=2
DP
40.4
DN
19.5
IgG - AF 488
IgA - AF 647
Sample Name
VH10 - T=3
DP
94.7
IgG - AF 488
IgA - AF 647
Sample Name
VH11 - T=1
DP
28.4
DN
29.8
IgG - AF 488
IgA - AF 647
Sample Name
VH11 - T=2
DP
95.1
IgG - AF 488
IgA - AF 647
Sample Name
VH11 - T=3
DP
96.4
IgG - AF 488
IgA - AF 647
Sample Name
VH12 - T=1
DP
92.9
DN
1.12
IgG - AF 488
IgA - AF 647
Sample Name
VH12 - T=2
DP
95.0
IgG - AF 488
IgA - AF 647
Sample Name
VH12 - T=3
DP
17.6
DN
41.3
IgG - AF 488
IgA - AF 647

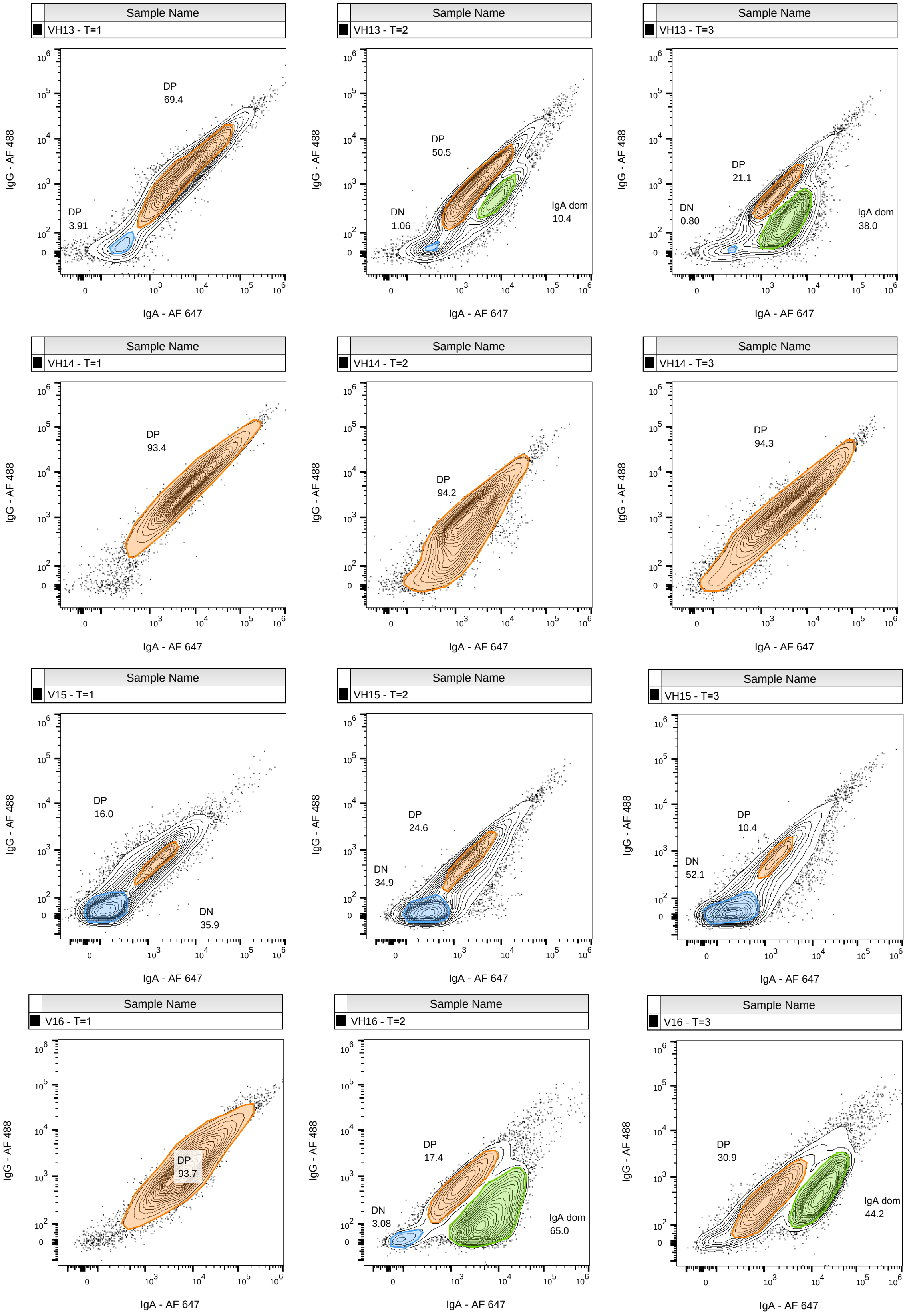
Sample Name
VH13 - T=1
DP
69.4
DP
3.91
IgG - AF 488
IgA - AF 647
Sample Name
VH13 - T=2
DP
50.5
DN
1.06
IgA dom
10.4
IgG - AF 488
IgA - AF 647
Sample Name
VH13 - T=3
DP
21.1
DN
0.80
IgA dom
38.0
IgG - AF 488
IgA - AF 647
Sample Name
VH14 - T=1
DP
93.4
IgG - AF 488
IgA - AF 647
Sample Name
VH14 - T=2
DP
94.2
IgG - AF 488
IgA - AF 647
Sample Name
VH14 - T=3
DP
94.3
IgG - AF 488
IgA - AF 647
Sample Name
V15 - T=1
DP
16.0
DN
35.9
IgG - AF 488
IgA - AF 647
Sample Name
VH15 - T=2
DP
24.6
DN
34.9
IgG - AF 488
IgA - AF 647
Sample Name
VH15 - T=3
DP
10.4
DN
52.1
IgG - AF 488
IgA - AF 647
Sample Name
V16 - T=1
DP
93.7
IgG - AF 488
IgA - AF 647
Sample Name
VH16 - T=2
DP
17.4
DN
3.08
IgA dom
65.0
IgG - AF 488
IgA - AF 647
Sample Name
V16 - T=3
DP
30.9
IgA dom
44.2
IgG - AF 488
IgA - AF 647

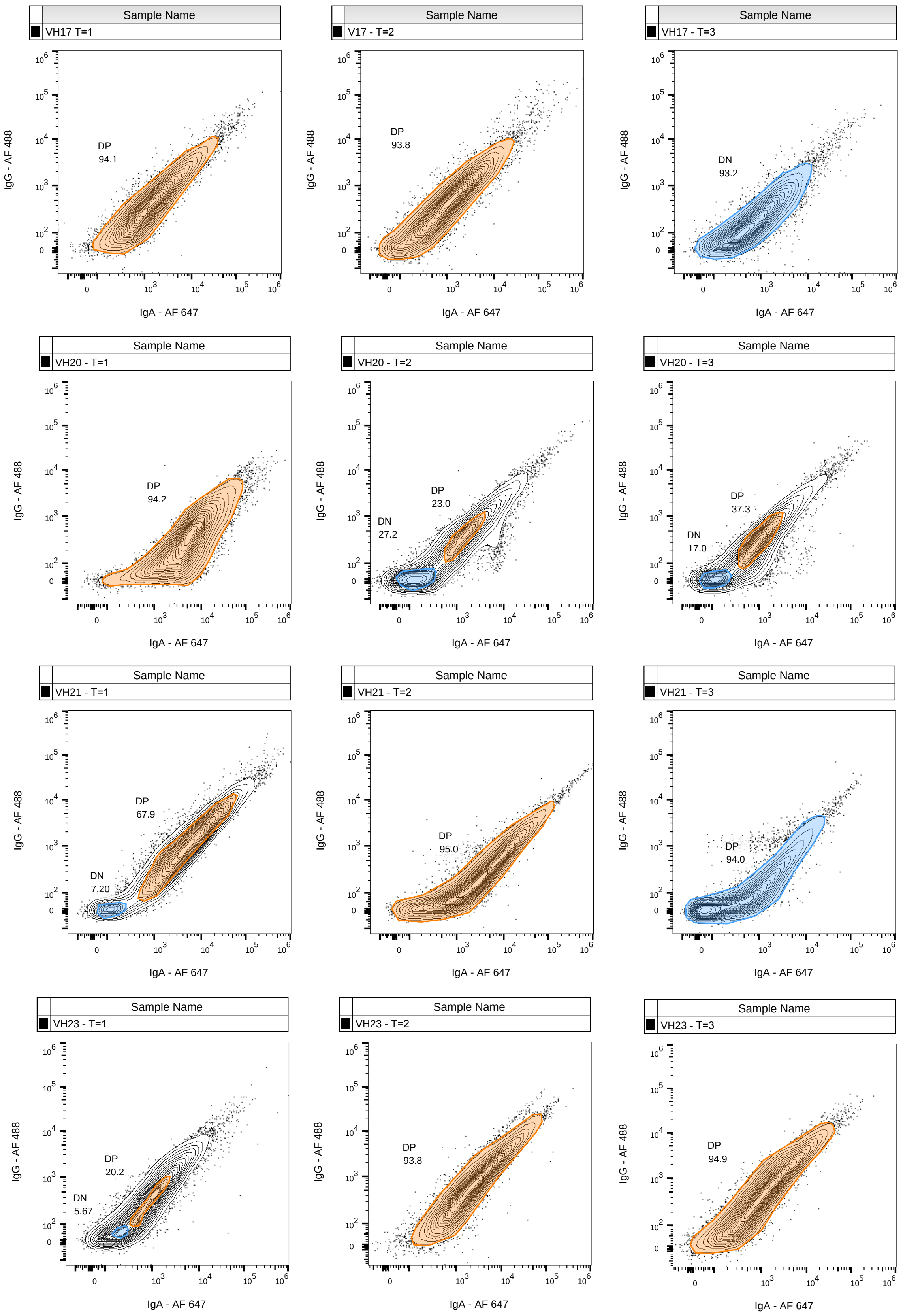

Sample Name
VH17 T=1
DP
94.1
Sample Name
V17 - T=2
DP
93.8
Sample Name
VH17 - T=3
DN
93.2
Sample Name
VH20 - T=1
DP
94.2
Sample Name
VH20 - T=2
DP
23.0
DN
27.2
Sample Name
VH20 - T=3
DP
37.3
DN
17.0
Sample Name
VH21 - T=1
DP
67.9
DN
7.20
Sample Name
VH21 - T=2
DP
95.0
Sample Name
VH21 - T=3
DP
94.0
Sample Name
VH23 - T=1
DP
20.2
DN
5.67
Sample Name
VH23 - T=2
DP
93.8
Sample Name
VH23 - T=3
DP
94.9
IgG - AF 488
IgA - AF 647

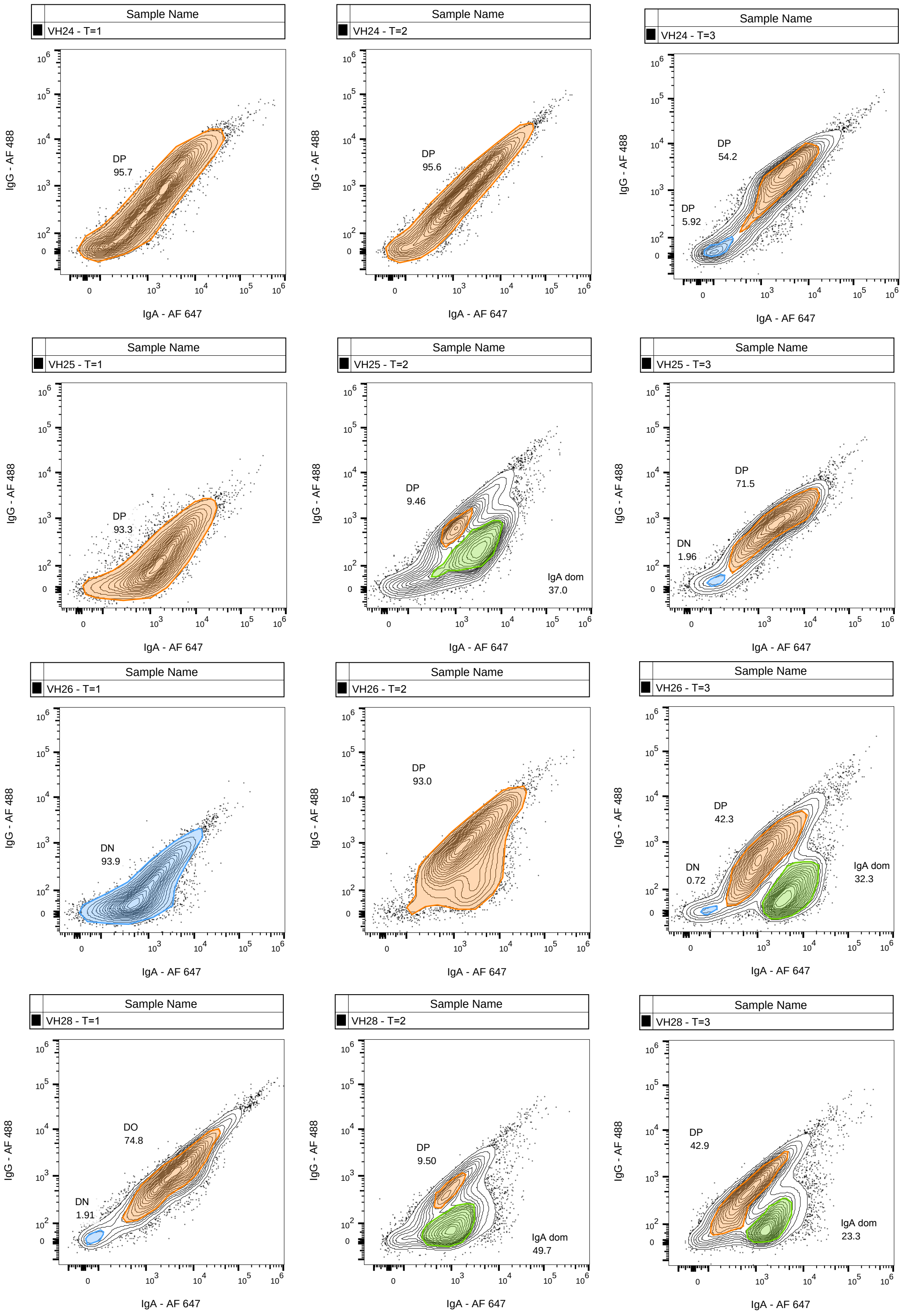
Sample Name
VH24 - T=1
DP
95.7
Sample Name
VH24 - T=2
DP
95.6
Sample Name
VH24 - T=3
DP
54.2
DP
5.92
Sample Name
VH25 - T=1
DP
93.3
Sample Name
VH25 - T=2
DP
9.46
IgA dom
37.0
Sample Name
VH25 - T=3
DP
71.5
DN
1.96
Sample Name
VH26 - T=1
DN
93.9
Sample Name
VH26 - T=2
DP
93.0
Sample Name
VH26 - T=3
DP
42.3
DN
0.72
IgA dom
32.3
Sample Name
VH28 - T=1
DO
74.8
DN
1.91
Sample Name
VH28 - T=2
DP
9.50
IgA dom
49.7
Sample Name
VH28 - T=3
DP
42.9
IgA dom
23.3
IgG - AF 488
IgA - AF 647

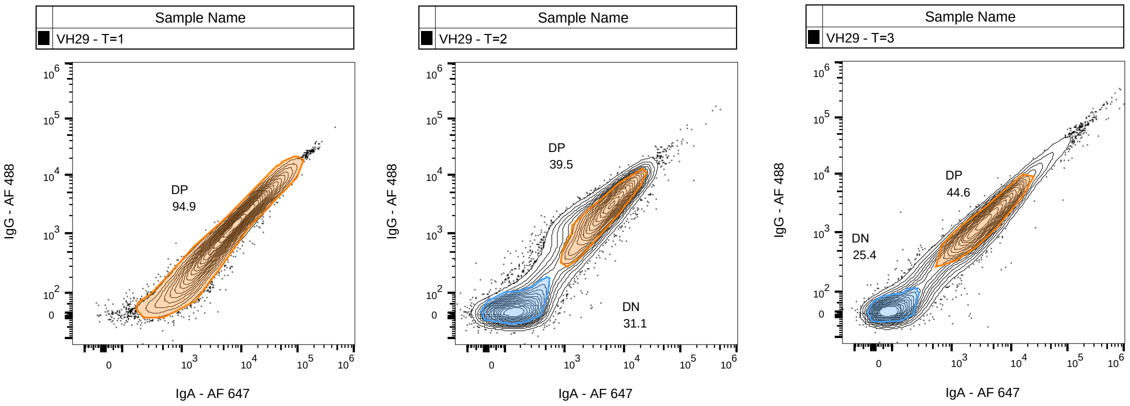
Sample Name
VH29 - T=1
DP
94.9
IgG - AF 488
IgA - AF 647
Sample Name
VH29 - T=2
DP
39.5
DN
31.1
IgG - AF 488
IgA - AF 647
Sample Name
VH29 - T=3
DP
44.6
DN
25.4
IgG - AF 488
IgA - AF 647

Table SM2. Percentages and MFI values of the present populations

ID	time point	bacterial dominance	DP %	DP IgA MFI	DP IgG MFI	DP IgA/IgG	IgAD %	IgAD IgA MFI	IgAD IgG MFI	IgAD IgA/IgG	DN %	DN IgA MFI	DN IgG MFI
VH01	1	*L. crispatus*	83,8	9305	6946	1,3					5,99	93	16,6
VH01	2	*L. crispatus*	36,9	1089	1337	0,8	17,5	9177	2257	4,1	7,04	112	26,8
VH01	3	*L. crispatus*	1,98	3319	2137	1,6	53,1	14590	1887	7,7	0,7	153	20,3
VH02	1	*L. crispatus*	93,2	6147	4103	1,5							
VH02	2	*L. crispatus*	93,5	1098	1579	0,7							
VH02	3	*L. crispatus*	81,4	1583	2862	0,6					2,32	231	42
VH03	1	non-*L. crispatus*	60,5	689	2673	0,3					0,44	117	44,9
VH03	2	non-*L. crispatus*	32,5	1410	1241	1,1					40,8	144	34,4
VH03	3	non-*L. crispatus*	92,1	1348	1351	1,0							
VH04	1	*L. crispatus*	58	8597	2332	3,7					30,7	88,4	6,1
VH04	2	*L. crispatus*	81,4	4250	258	16,5					1,86	130	23,8
VH04	3	*L. crispatus*	10,1	1509	689	2,2					47,9	161	20,8
VH05	1	*L. crispatus*	37,4	1701	563	3,0					29,1	97,7	14,2
VH05	2	*L. crispatus*	35,5	7343	859	8,5					37,3	105	7,08
VH05	3	*L. crispatus*	94,8	1696	288	5,9							
VH06	1	*L. crispatus*	75	8201	3015	2,7					4,82	92,8	13
VH06	2	*L. crispatus*	18,8	1545	409	3,8	36,6	2328	76,7	30,4	1,18	119	11
VH06	3	*L. crispatus*	61,8	3685	793	4,6					5,65	103	15,4
VH07	1	non-*L. crispatus*	47,2	3083	1119	2,8					25,4	171	6,1
VH07	2	non-*L. crispatus*									93,9	480	97,8
VH07	3	non-*L. crispatus*	65,2	4759	548	8,7					5,31	127	12,5
VH08	1	non-*L. crispatus*	64,3	2654	1107	2,4					2,16	172	30,1
VH08	2	non-*L. crispatus*	95	2048	185	11,1							
VH08	3	non-*L. crispatus*	56,1	1920	251	7,6					1,28	211	14,4
VH09	1	*L. crispatus*	55,7	3956	1125	3,5					5,67	140	13,5
VH09	2	*L. crispatus*	9,81	1286	334	3,9	32,3	878	59	14,9			
VH09	3	*L. crispatus*	20,8	2696	918	2,9	26,9	2696	234	11,5	2,02	113	20,1
VH10	1	non-*L. crispatus*	95,3	4683	501	9,3							
VH10	2	non-*L. crispatus*	40,4	1940	379	5,1					19,5	169	7,57
VH10	3	non-*L. crispatus*	94,7	1712	505	3,4							
VH11	1	*L. crispatus*	28,4	1802	511	3,5					29,8	269	32,1
VH11	2	*L. crispatus*	95,1	1221	159	7,7							
VH11	3	*L. crispatus*	96,4	1116	230	4,9							
VH12	1	non-*L. crispatus*	92,9	8825	3664	2,4					1,12	107	9,04
VH12	2	non-*L. crispatus*	95	2416	1095	2,2							
VH12	3	non-*L. crispatus*	17,6/41,3*	16364/701	4494/277	3,6/2,5							
VH13	1	*L. crispatus*	69,4	5593	1644	3,4					3,91	299	39,7
VH13	2	*L. crispatus*	50,5	2257	707	3,2	10,4	8254	517	16,0	1,06	341	28,3
VH13	3	*L. crispatus*	21,1	2161	642	3,4	38	3536	172	20,6	0,8	267	20,1
VH14	1	*L. crispatus*	93,4	6516	3969	1,6							
VH14	2	*L. crispatus*	94,2	1618	646	2,5							
VH14	3	*L. crispatus*	94,3	3283	1362	2,4							
VH15	1	non-*L. crispatus*	16	1534	566	2,7					35,9	151	35,7
VH15	2	non-*L. crispatus*	24,6	1834	563	3,3					34,9	320	19,6
VH15	3	non-*L. crispatus*	10,4	1664	761	2,2					52,1	236	20,8

VH16	1	*L. crispatus*	93,7	7200	1322	5,4							
VH16	2	L. crispatus	17,4	1562	626	2,5	65	5793	122	47,5	3,08	111	16,4
VH16	3	L. crispatus	30,9	1065	315	3,4	44,2	15569	450	34,6			
VH17	1	non-*L. crispatus*	94,1	1227	362	3,4							
VH17	2	non-*L. crispatus*	93,8	846	343	2,5							
VH17	3	non-*L. crispatus*									93,2	643	150
VH20	1	non-*L. crispatus*	94,2	4900	262	18,7							
VH20	2	non-*L. crispatus*	23	1312	372	3,5					27,2	149	12,7
VH20	3	non-*L. crispatus*	37,3	943	307	3,1					17	127	12,4
VH21	1	non-*L. crispatus*	67,9	4465	899	5,0					7,2	124	7,77
VH21	2	non-*L. crispatus*	95	2385	168	14,2							
VH21	3	non-*L. crispatus*									94	452	45,2
VH23	1	non-*L. crispatus*	20,2	867	332	2,6					5,67	244	55,9
VH23	2	non-*L. crispatus*	93,8	1926	529	3,6							
VH23	3	non-*L. crispatus*	94,9	916	375	2,4							
VH24	1	non-*L. crispatus*	95,7	1150	412	2,8							
VH24	2	non-*L. crispatus*	95,6	1180	491	2,4							
VH24	3	non-*L. crispatus*	54,2	2824	1892	1,5					5,92	131	36
VH25	1	*L. crispatus*	93,3	1649	124	13,3							
VH25	2	*L. crispatus*	9,46	1008	619	1,6	37	2805	219	12,8			
VH25	3	*L. crispatus*	71,5	2327	761	3,1					1,96	139	32,8
VH26	1	*L. crispatus*									93,9	779	72,7
VH26	2	*L. crispatus*	93	2012	697	2,9							
VH26	3	*L. crispatus*	42,3	1250	491	2,5	32,3	4867	75,8	64,2	0,64	100	11,4
VH28	1	*L. crispatus*	74,8	3907	1002	3,9					1,91	68,6	19,9
VH28	2	*L. crispatus*	9,5	885	501	1,8	49,7	989	66,5	14,9			
VH28	3	*L. crispatus*	42,9	600	412	1,5	23,3	1814	87	20,9			
VH29	1	*L. crispatus*	94,9	4874	785	6,2							
VH29	2	*L. crispatus*	39,5	4716	2309	2,0					31,1	176	24,3
VH29	3	*L. crispatus*	44,6	3338	1372	2,4					25,4	102	20,8

DP = Double positive population, IgAD = IgA dominant population, DN = double negative population. MFI = Median Fluorescence Intensity. Mean IgA MFI + 3 standard deviation from no stain samples: 845.3. Mean IgG MFI + 3 standard deviations from no stain samples: 171, 5. *sample with two double positive populations, population 1/population 2
